# The research potential of A20 in psoriatic arthritis

**DOI:** 10.3389/fimmu.2025.1630198

**Published:** 2026-01-09

**Authors:** Yixuan Wang Wan, Xiaoru Duan, Zilin Jin, Hongxiang Chen

**Affiliations:** 1 Department of Dermatology, Union Hospital, Tongji Medical College, Huazhong University of Science and Technology, Wuhan, China; 2 Department of Rheumatology and Immunology, Union Hospital, Tongji Medical College, Huazhong University of Science and Technology, Wuhan, China; 3 Department of Dermatology, Zhongnan Hospital of Wuhan University, Wuhan University, Wuhan, China; 4 Department of Dermatology, Shenzhen Nanshan People’s Hospital, Shenzhen, China

**Keywords:** psoriatic arthritis, A20, animal model, cell death, immune response

## Abstract

Psoriasis, a systemic inflammatory disorder, extends beyond its classical dermatological presentation to encompass multiple manifestations including arthritis, inflammatory bowel disease, ocular inflammation (conjunctivitis/uveitis), and cardiovascular manifestations such as aortic valve pathology. While cutaneous manifestations have been extensively characterized, psoriatic arthritis (PsA) remains challenging to investigate, primarily due to the paucity of suitable experimental models that accurately recapitulate human disease. The ubiquitin-editing enzyme A20 (encoded by TNFAIP3) emerges as a critical regulatory molecule, serving dual functions in suppressing NF-κB signaling pathways and modulating programmed cell death mechanisms. Genome-wide association studies have established TNFAIP3 polymorphisms as susceptibility loci for both psoriasis and PsA. Murine models with A20 deficiencies demonstrate spontaneous development of cutaneous psoriasiform lesions and articular inflammation, with genetic manipulation techniques generating diverse mutation patterns that manifest in heterogeneous phenotypes. Systematic analysis of these preclinical models offers valuable insights into the molecular pathogenesis of PsA, potentially bridging current knowledge gaps in disease mechanisms and therapeutic target identification.

## 1 Introduction

Psoriasis is a chronic systemic inflammatory disorder that affects 2%–4% of the global population and presents with hallmark dermatological features, including erythematous plaques, silvery desquamation, and the Auspitz sign (1, 2). Emerging evidence underscores its systemic nature, with multiorgan manifestations affecting articular structures (particularly axial and peripheral joints), cardiovascular systems, ocular tissues, and gastrointestinal tract (1, 2). Of particular clinical significance, psoriatic arthritis (PsA), manifesting both psoriasis and arthropathy, develops in 20%–30% of psoriasis patients, typically emerging within a decade of initial dermatological diagnosis in 85% of cases (3). PsA affects patients through physical, psychological, socioeconomic burdens, and especially irreversible joint destruction when treated unproperly (4). The pathophysiology arises from intricate interaction between polygenic susceptibility and environmental precipitants, leading to a pathological process that often extends beyond skin and joint involvement (1). Recent advances in genetic epidemiology studies have delineated shared susceptibility loci between psoriasis and PsA, such as HLA-B/C, IL23A, IL23R, IL12B, and REL (1). Notably, the ubiquitin-modifying enzyme A20, encoded by the TNFAIP3 gene, has emerged as a critical regulator of PsA pathogenesis, with substantial evidence derived from human genetic studies (3, 5, 6). Genome-wide association studies (GWAS) have consistently identified polymorphisms in the TNFAIP3 locus as susceptibility factors not only for psoriasis vulgaris but also specifically for PsA, highlighting its distinct genetic contribution to joint involvement (7–9). This genetic predisposition is functionally consequential, as recent single-cell RNA sequencing of circulating immune cells from PsA patients revealed dysregulated expression of TNFAIP3 and related genes, suggesting its role as a potential biomarker for distinguishing PsA from cutaneous psoriasis (10). Furthermore, the clinical relevance of A20 is underscored by pharmacogenomic studies, which indicate that specific TNFAIP3 variants can predict therapeutic response to anti-TNF agents, a mainstay of PsA treatment (11, 12). The convergence of genetic, transcriptional, and pharmacogenetic evidence from human subjects positions A20 as a key molecular hub in PsA, warranting in-depth mechanistic investigation.

To bridge these human genetic findings with mechanistic insights, experimental models—such as those employing epidermal-specific A20 knockout mice—have been developed. These models have successfully recapitulated key PsA phenotypes, providing a systems-level platform to elucidate A20’s dual role in cutaneous and articular inflammation. Nevertheless, emerging evidence reveals substantial heterogeneity across existing preclinical models, necessitating systematic evaluation of their translational relevance. This review critically synthesizes current knowledge from A20-deficient models, elucidating their contributions to understanding PsA pathogenesis while addressing critical gaps in phenotypic characterization across experimental systems. Through this analysis, we aim to establish a framework for optimizing translational research strategies in psoriatic disease.

## 2 PsA: pathophysiological and diagnostic insights with focus on animal models

### 2.1 Classification challenges and implications for modeling

PsA represents a complex disorder that intersects both dermatological and rheumatological fields (13, 14). Its dual classification—as a comorbidity of psoriasis and a spondyloarthritis subtype with psoriatic skin lesions—reflects distinct mechanistic pathways that challenge disease modeling (14). Animal models must address this nosological complexity by replicating the unique features of PsA, which are elaborated subsequently.

### 2.2 Cutaneous-ungual pathology as a modeling prerequisite

Cutaneous and ungual pathologies are essential prerequisites for modeling PsA, as they underpin diagnostic validity and biological relevance. Approximately 75%-84% of PsA patients exhibit psoriatic skin lesions and 80% present with nail lesions (15, 16). Diagnostic frameworks (e.g., Moll and Wright, Bennett and Vasey-Espinoza criteria) require the presence of psoriatic skin or nail lesions for definitive PsA classification (17). Additionally, dactylitis, observed in nearly half of PsA cases, further complements these phenotypes in prognostic and disease progression assessments (14). The CASPAR classification criteria, which are the most widely used currently, list key features including evidence of psoriasis, psoriatic nail dystrophy, and dactylitis, with at least one being required for a diagnosis of PsA (18). The integration of dermal or ungual pathology in PsA animal models is critical to ensure translational fidelity, as their absence compromises the alignment with human diagnostic paradigms and obscures insights into disease pathogenesis. These clinical hallmarks directly validate model relevance, bridging experimental systems to the complex pathophysiology of PsA.

### 2.3 Heterogeneity in articular involvement

PsA demonstrates marked heterogeneity in joint involvement, complicating differentiation from RA and OA (19, 20). In animal models, paw swelling is a common but nonspecific indicator of arthritis. Pathological features such as synovitis, bone erosion, and new bone formation can help assess the severity of arthritis but are insufficient to confirm PsA. Although it is said that PsA involves the distal interphalangeal joints (DIPs), and RA involves proximal interphalangeal joints (PIPs), inflammation of both DIPs and PIPs can occur in PsA patients (19–21). Therefore, both DIP and PIP inflammation can be taken as features of a PsA animal model. Additionally, while RA typically presents with symmetric arthritis, PsA can manifest as symmetric or asymmetric, oligo- or polyarticular arthritis, making it hard to identify PsA models through these traits.

### 2.4 Enthesitis as a central mechanism in PsA model development

**Figure 1:**
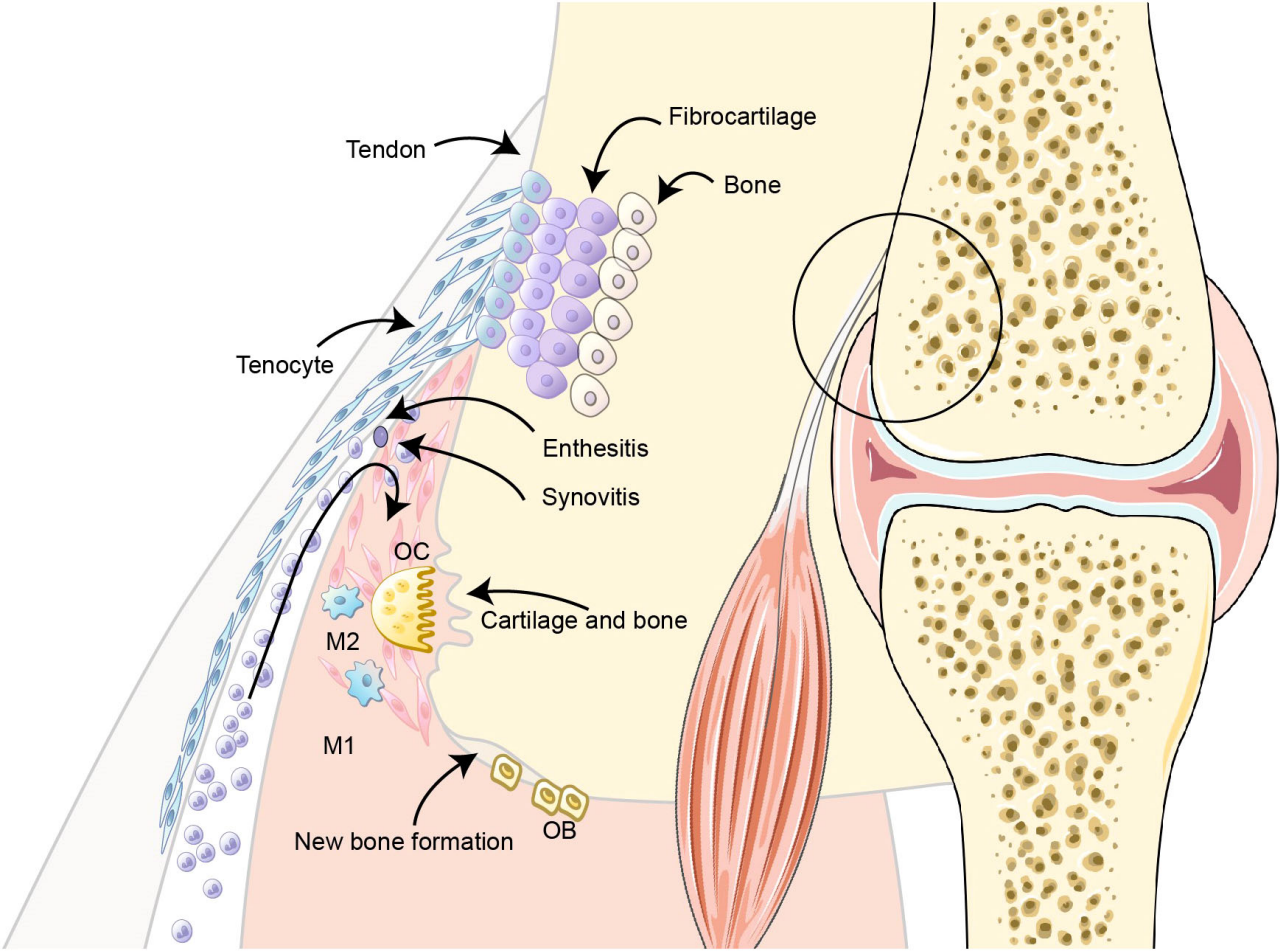
Pathological progression of PsA at the enthesis and adjacent joint. The enthesis, the site of tendon/ligament insertion into bone, comprises four histologically distinct zones: fibrous connective tissue, uncalcified fibrocartilage, calcified fibrocartilage, and subchondral bone. In PsA, enthesitis initiates at the enthesis and propagates to the synovium, culminating in synovitis. Sustained inflammation facilitates cartilage degradation and bone invasion. Pathological osteoclast (OC) activation coupled with dysregulated osteoblast (OB) activity disrupts bone remodeling equilibrium, manifesting as concurrent bone erosion and aberrant neoformation. This dual-process mechanism underlies the characteristic structural damage observed in PsA. OC, osteoclast; OB, osteoblast; M1, Classical activated macrophages; M2, alternatively activated macrophages.

Enthesitis, the inflammation at ligament/tendon insertion sites, is a pathognomonic feature distinguishing PsA from RA (22). Unlike RA, where inflammation originates at synovium, pathology in PsA is thought to begin at entheses (23) (**Figure 1**). Studies have found early activation of synovio-entheseal fibroblast at weight-bearing sites in subclinical PsA patients and mouse models, indicating the role of mechano-inflammation in PsA (24, 25). Therefore, it is essential to appraise enthesitis when evaluating PsA animal models. Enthesitis manifests not only in peripheral, but also in axial in PsA (26). The presence of axial pathologies such as sacroiliitis and spondylitis underscores the need for experimental models that integrate spinal entheseal inflammation with synovial involvement for comprehensive pathophysiological analysis.

### 2.5 Osteoproliferation: an imaging-based diagnostic criterion for PsA

Osteoproliferation refers to the aberrant formation of new bone tissue and is a hallmark feature of PsA (27). Osteoproliferative lesions in PsA, such as enthesophytes in the Achilles tendon and syndesmophytes in axial structures, are pathologically associated with dysregulated immune activation (28). Unlike degenerative osteophytes, PsA-associated osteoproliferation is driven by inflammatory pathways, particularly the IL-23/IL-17 axis, which promotes mesenchymal stem cell differentiation and osteoblast activation (27, 29). As osteoproliferation constitutes the only imaging feature included in the CASPAR criteria, its manifestation in preclinical mouse models represents a critical validation benchmark for their translational relevance.

### 2.6 Seronegativity and biomarker discovery in model validation

While the majority of SpA patients exhibit seronegativity for rheumatoid factor (RF), approximately 10% of individuals with uncomplicated psoriasis and 15% of healthy individuals demonstrate RF positivity (30, 31). Other serological markers, including cyclic citrullinated peptide (CCP) antibodies and antinuclear antibodies (ANA), may reflect systemic autoimmune activation but lack diagnostic specificity for PsA (31). This evidence supports the adjunctive role of serological biomarkers in refining validation criteria for PsA animal models.

Developing experimental animal models for PsA is significantly hindered by the disease’s complex pathophysiology and heterogeneous clinical manifestations. To be effective, such models must faithfully recapitulate key features like skin-joint crosstalk, entheseal inflammation, and variable joint involvement. Beyond this phenotypic challenge, the field must also translate pathophysiological understanding into identifiable targets. Here, human genetic studies have highlighted critical regulatory molecules. Among these, the A20 protein, encoded by the TNFAIP3 susceptibility locus shared by psoriasis and PsA, stands out (3). Given its specific link to both cutaneous and articular disease and its master role in curbing inflammation, A20 emerges as a compelling molecular bridge for deciphering PsA mechanisms and a potential candidate for biomarker development (11, 12).

## 3 Structural and functional insights into A20

**Figure 2:**
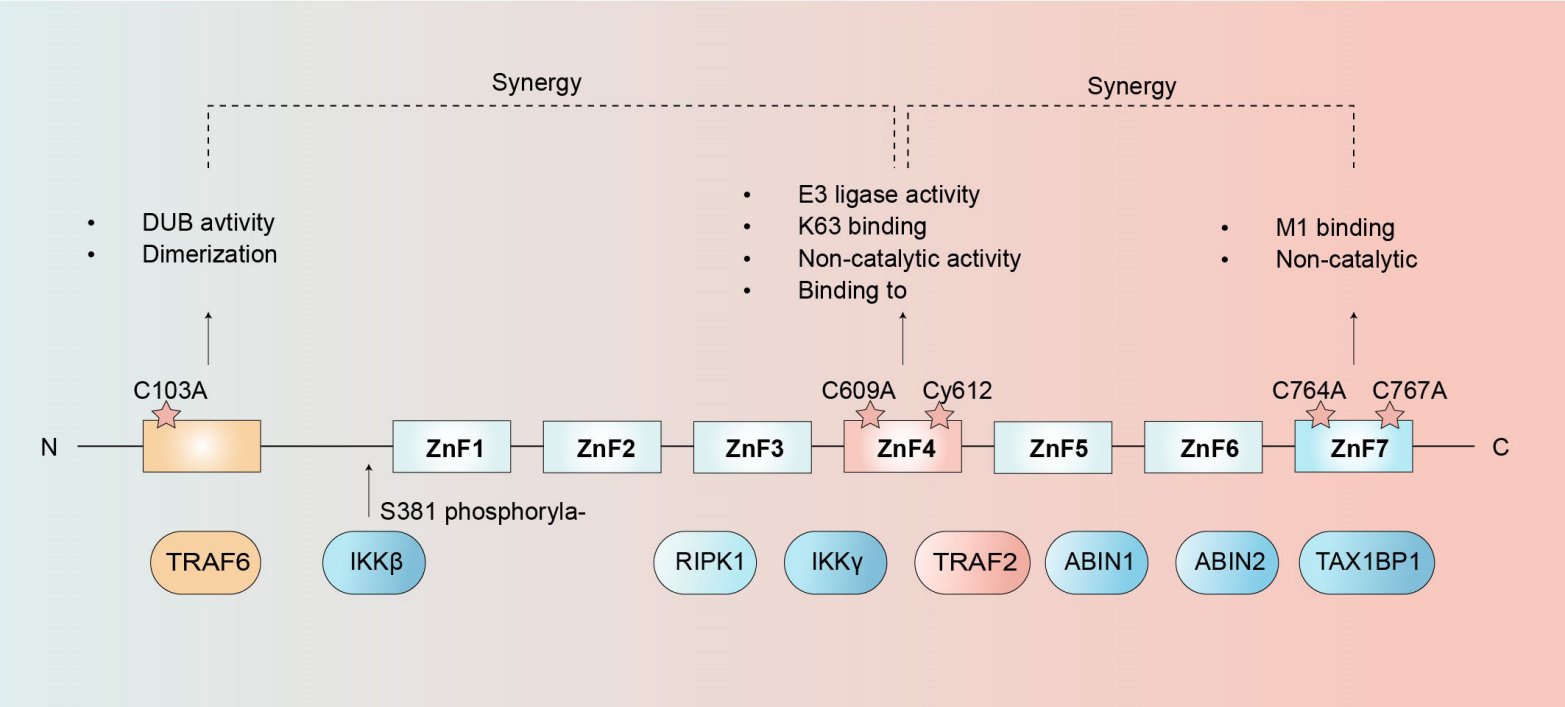
Structural and functional domains of A20. The N-terminal ovarian tumor (OTU) domain is responsible for deubiquitinating enzyme (DUB) activity. At the C-terminus, seven zinc finger (ZnF) domains are present, with ZnF4 and ZnF7 synergistically contributing to DUB activity. ZnF4 specifically binds E2 ubiquitin-conjugating enzymes and mediates E3 ubiquitin ligase activity. Additionally, A20 selectively interacts with K63-linked ubiquitin chains through ZnF4 and M1-linked ubiquitin chains via ZnF7. Both ZnF4 and ZnF7 domains further participate in non-catalytic regulatory functions of A20.

A20, encoded by the TNFAIP3 gene, is a ubiquitin-editing enzyme with potent anti-inflammatory and anti-apoptotic functions, primarily through its regulation of NF-κB signaling and cell death pathways (32). Structurally, A20 consists of an N-terminal ovarian tumor (OTU) domain and a C-terminal region with seven zinc finger (ZnF) domains, each mediating distinct functional roles (**Figure 2**). The OTU domain catalyzes deubiquitination of M1-, K63-, and K48-linked polyubiquitin chains, whereas ZnF4 operates as an E3 ligase, driving K48-linked polyubiquitination and subsequent proteasomal degradation of substrates. ZnF4 further interacts with E2 enzymes (e.g., Ubc13 and UbcH5c) to suppress associated E3 ligase activity (33, 34). Dimerization of A20 promotes synergistic coordination between domains, amplifying regulatory effects (32, 35). Additionally, ZnF4 collaborates with ZnF7 to bind M1-linked ubiquitin chains, thereby inhibiting LUBAC-mediated NF-κB activation. The ZnF7 domain is recruited by the TNFR1 and RANK complexes via M1 chain, competitively displacing other ubiquitin-binding proteins to prevent the degradation of the M1 chain (36).

### 3.1 NF-κB pathway is restricted by A20

NF-κB, a transcription factor present in all nucleated cells, governs critical processes including cell survival and inflammatory responses (37). Its dysregulated activation is implicated in the pathogenesis of autoimmune disorders and chronic inflammatory diseases (38). Both *in vivo* and *in vitro* evidence has indicated that A20 inhibits the transcription of NF-κB response genes (39). This inhibition effect is realized through protein-protein interactions, without affecting its nuclear translocation or DNA binding (39, 40). Notably, A20’s deubiquitinating (DUB) activity is dispensable for NF-κB regulation, highlighting its non-catalytic roles (35).

**Figure 3:**
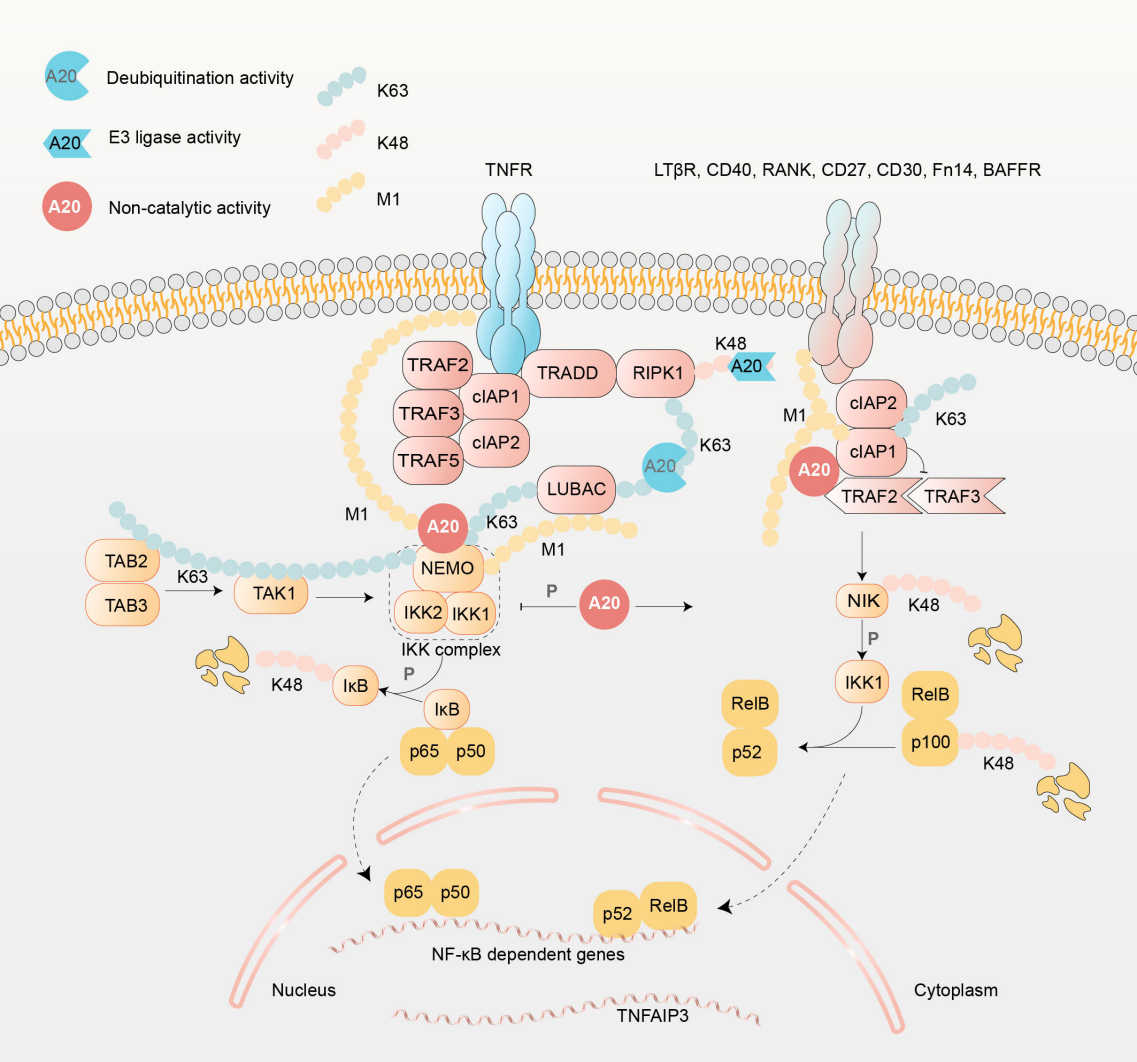
Regulatory mechanisms of A20 in canonical and non-canonical NF-κB signaling pathways. In the canonical pathway, activation of IL-1Rs, TLRs, TNFR, TCRs, or BCRs triggers recruitment of adaptor proteins to the membrane, leading to modulation of the IKK complex. Following TNFR1 stimulation, a signaling complex comprising TRADD, TRAF2, TRAF5, cIAP1/2, and RIPK1 assembles via K63- and M1-linked ubiquitin chains (41). This complex undergoes post-translational regulation by LUBAC, OTULIN, CYLD, and A20, forming a platform for TAK/TAB and IKK complex (IKK1, IKK2, NEMO) recruitment (39). Upon stimulation, A20 is recruited within 15-30minutes, and remains bound to IKKγ for at least 2 hours via its ZnF4 and ZnF7 domains (39). Furthermore, A20’s binding to NEMO or TAB2 concurrently suppresses IKK phosphorylation (40, 42). LUBAC-mediated NEMO ubiquitination (via K63- and M1-linked chains) promotes IKK activation, enhancing NF-κB-driven c-Jun phosphorylation (43, 44). A20 counteracts this process non-catalytically by competing with LUBAC for ubiquitin chain binding through ZnF4 and ZnF7, thereby attenuating NF-κB activation (45, 46). Additionally, A20 interacts with TRADD, TRAF2, and RIPK1, though TRAF2 binding is dispensable for NF-κB regulation (40, 42, 47, 48). In the non-canonical pathway, A20 stabilizes NIK via its ZnF7 domain (40). TNFR engagement recruits cIAP1/2, TRAF2, and TRAF3 to form a NIK ubiquitin ligase complex (40). A20 disrupts TRAF2/TRAF3 interactions by binding cIAP1, either directly or indirectly, preventing NIK degradation and enabling its accumulation (43). This stabilizes NIK, which activates IKK1 to process p100 into p52, driving non-canonical NF-κB signaling (43). A20 deficiency abrogates NIK stabilization and p100 processing, redirecting signaling toward the canonical pathway (43). Notably, canonical pathway-derived K63-linked ubiquitin chains may competitively inhibit A20-cIAP1/2 interactions, suppressing non-canonical activation (43).

NF-κB can be activated through two pathways, a canonical and a non-canonical one. Here we show the molecular mechanism of TNFR1 signaling as an example (**Figure 3**). A20 acts as a molecular switch between the two pathways, suppressing the pro-inflammatory canonical pathway while promoting the non-canonical pathway, which is considered less harmful (49). This shift dampens NF-κB activation, thereby slowing and mitigating inflammatory responses.

The interplay between A20 and NF-κB forms a negative feedback loop. A20 not only restricts NF-κB activity but is also reciprocally induced by NF-κB through transient cytoplasmic expression mechanisms in response to proinflammatory stimuli, such as TNF, IL-1, LPS, CD40 engagement, or viral proteins (e.g., HTLV-1 Tax and EBV LMP1) (40). However, basal A20 expression has also been detected in unstimulated cells, suggesting additional homeostatic functions beyond stimulus-responsive regulation (50). This multilayered control underscores A20’s critical role in balancing NF-κB-mediated immune activation and inflammatory pathology.

### 3.2 Cell death pathways are regulated by A20

**Figure 4:**
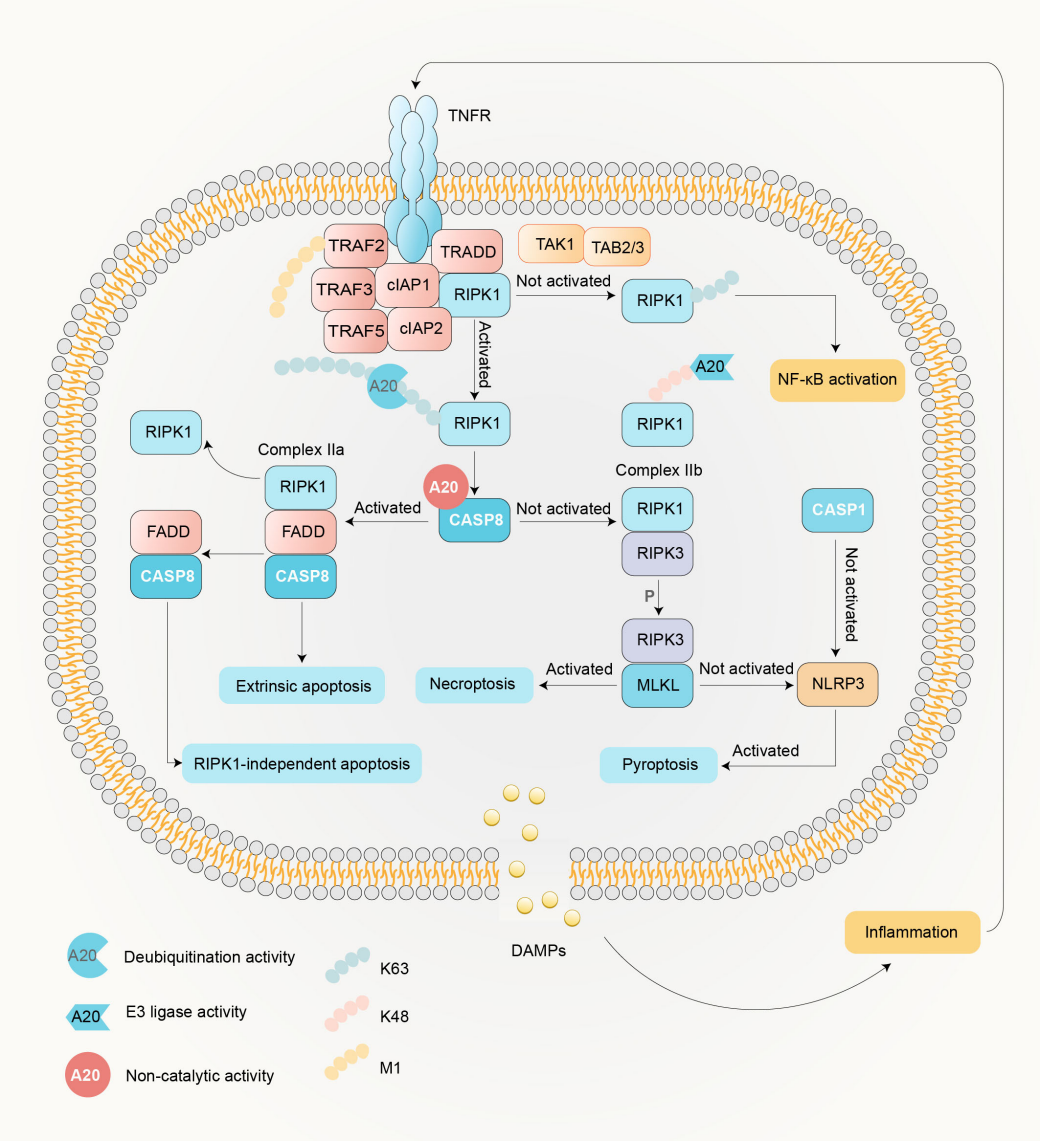
Regulatory Dynamics of A20 in Cell death. The inflammatory cytokine TNF-α initiates TNFR1 signaling by binding to its receptor, triggering the assembly of Complex I (comprising TRADD, TRAF2/5, cIAP1/2, and RIPK1) within five minutes (39). Post-translational modifications—including polyubiquitination, deubiquitination, and phosphorylation—dictate RIPK1 activation status. Non-activated RIPK1 perpetuates canonical NF-κB signaling, whereas activated RIPK1 bifurcates into two distinct pathways contingent on caspase-8 activity (12, 13). Caspase-8 activation enables RIPK1 to assemble Complex IIa (ripoptosome) with FADD, initiating caspase-3/-7 cascades that drive RIPK1-dependent or -independent apoptosis (51). Conversely, caspase-8 inhibition redirects RIPK1 to form Complex IIb (necrosome) with RIPK3, leading to MLKL phosphorylation (52). Activated MLKL oligomerizes to permeabilize plasma membranes, executing necroptosis. In scenarios where MLKL is not activated, NLRP3 inflammasome assembly ensues, particularly in bone marrow-derived macrophages (BMDMs) and intestinal epithelial cells, culminating in IL-1β cleavage and GSDMD-mediated pyroptosis (53, 54). Notably, this inflammasome-dependent pathway is absent in skin (55, 56).

When an organism encounters threats such as trauma, infection, and stress, implicated cells may receive death signals and induce a sequence of molecular events within the cell. Cell death pathways exhibit complex interplay with the NF-κB signaling cascade, with their regulation being mediated by A20 (47) (**Figure 4**). The relationship between cell death and NF-κB activation represents a dynamic equilibrium rather than a transient interaction. Experimental evidence from A20-deficient murine models demonstrates that TNF-α stimulation induces imbalance in this regulatory system, resulting in dyshomeostasis of NF-κB signaling and pathological activation of cell death pathways (47).

Functionally, A20 operates through both enzymatic activity and structural interactions to determine cellular fate. For instance, A20’s OTU domain removes K63-linked ubiquitin chains from RIPK1, attenuating NF-κB signaling, while its ZnF4 and ZnF7 domain together promotes K48-linked ubiquitination, targeting RIPK1 for proteasomal degradation (35, 57). Additionally, A20 inhibits RIPK3-dependent necroptosis via its Cys103 residue in OTU domain, independent of ZnF4 activity (58). The ZnF7 domain of A20 is indispensable for inhibiting TNF-induced NF-κB activation and cell death (50). It facilitates the recruitment of A20 towards TNFR1 signaling complexes by combining M1-linked ubiquitin chains (57, 59). A20 thus stabilizes M1-linked ubiquitin chains in the complex, preventing the dissociation of RIPK1 and subsequent formation of complex II, which favors apoptosis or necroptosis (57, 59). This stabilization counteracts the effects of CYLD, a deubiquitinating enzyme that promotes apoptosis (60). Furthermore, A20 inhibits caspase-8 transcription, providing an additional layer of apoptosis regulation, and a predisposition for necroptosis and inflammasome activation (41, 61). These findings underscore the regulation effect of A20 in the switch between NF-κB and cell death pathways, which may explain the insufficiency of IKK2 knockout to relieve arthritis, but requires further inhibition of necroptosis and inflammasome pathway. These mechanisms collectively highlight A20’s pivotal role in balancing inflammatory signaling and cell survival, offering insights into its therapeutic potential for inflammatory and autoimmune diseases (62).

### 3.3 A20 inhibits IL-17, IL-1R and TLR signaling pathways

**Figure 5:**
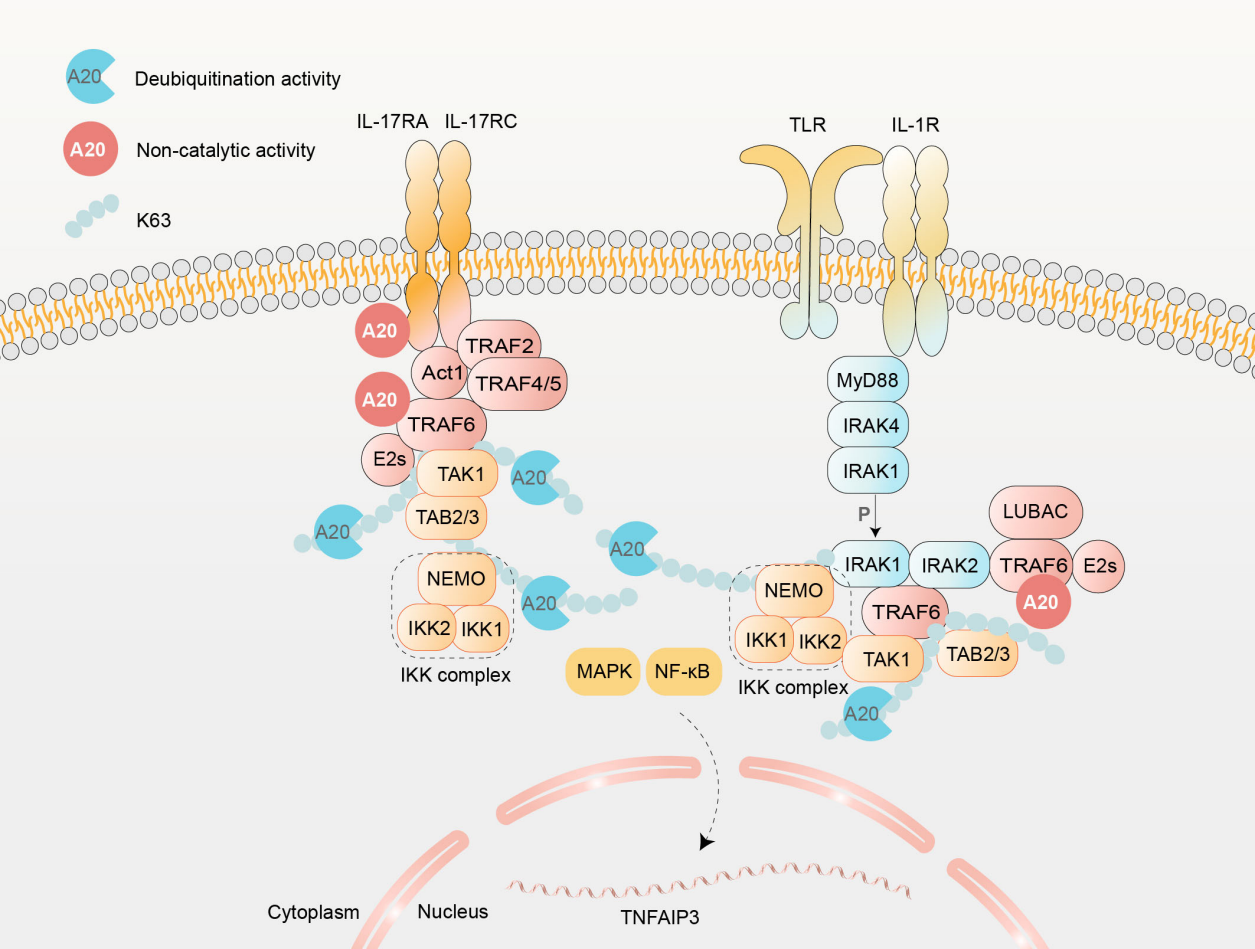
IL-17 and IL-1R/TLR Signaling Pathways regulated by A20. The binding of IL-17 induces heterodimerization of IL-17 receptors (IL-17Rs), which subsequently activates Act1 form the IL-17R-Act1 complex through its SEFIR domain (64). This complex recruits TRAFs, including TRAF2, TRAF4, TRAF5, and TRAF6, to mediate transcriptional and post-translational regulatory processes (65). In parallel, IL-1R or TLR activation initiates myeloid differentiation primary response 88 (MyD88) recruitment, facilitating interleukin-1 receptor-associated kinase (IRAK) 4 assembly. Phosphorylation of IRAK4 triggers IRAK1 activation and its subsequent dissociation from the receptor complex, enabling IRAK1 to interact with TRAF6 and propagate downstream signaling cascades. Across these pathways, TRAF6 undergoes K63-linked polyubiquitination, which involves E2 conjugating enzymes (E2s, such as Ubc13 and Uev1a) (65). A20 regulates this process catalytically by cleaving K63-lincked ubiquitin chains, and non-catalytically by inhibits the conjugation between TRAF6 and E2s. A20 further restricts IL-17 signaling by direct combination with CBAD domain of IL-17RA (64). K63-polyubiquitinated TRAF6 recruits TAK1, which associates with TAB2/3 and the IKK complex to activate NF-κB and MAPK (JNK, ERK, p38) pathways.

IL-17 receptor (IL-17R), IL-1 receptor (IL-1R), and Toll-like receptor (TLR) signaling pathways play pivotal roles in the pathogenesis of cutaneous and arthritic inflammation (35, 63) (**Figure 5**). These pathways induce A20 expression, which in turn exerts dual catalytic and non-catalytic inhibitory effects, thereby establishing an essential negative feedback regulatory loop (64). A20 suppresses inflammatory signaling via two distinct molecular strategies. First, its zinc finger 4 (ZnF4) domain disrupts the interaction between TRAF6 and E2 ubiquitin-conjugating enzymes (e.g., Ubc5 and Ubc13), promoting proteasomal degradation of these enzymes and thereby terminating pro-inflammatory signal transduction (34). Second, A20 cleaves K63-linked ubiquitin chains from critical signaling components (e.g., TRAF6 and NEMO) positioned downstream of pathway-specific adaptor proteins: Act1 in the IL-17 signaling cascade, and MyD88/IRAK in IL-1R/TLR-mediated pathways (66). Notably, A20 demonstrates additional pathway specificity through direct binding to the CBAD domain of IL-17RA, independent of TRAF6 or SEFIR domain involvement, further suppressing IL-17-driven inflammation (64). Functional validation comes from studies showing amplified TRAF6 recruitment to receptor complexes and heightened phosphorylation of IKK complex components and JNK in A20-deficient cellular models (33, 64).

### 3.4 A20 restricts JAK/STAT pathways

The JAK/STAT pathway is mediated by receptor-associated tyrosine kinases (JAKs) and transcription factors (STATs), facilitating rapid signal transduction from extracellular ligands to nuclear gene activation (67). A20, analogous to its regulatory roles in other inflammatory pathways, establishes a negative regulatory loop with STAT3 (68). Mechanistically, A20 interacts with STAT3 directly and suppresses the K63-ubiquitination of STAT3 (69). Studies have emphasized the role of JAK2-STAT3-A20 cascades in inflammation, especially the IL-6-driven inflammatory responses (68). Additionally, STAT1 hyperactivation in myeloid cells also contributes to aberrant immune responses in A20-deficient states (70).

These findings collectively establish A20 as a multifaceted regulator that constrains hyperactivation of critical inflammatory pathways through coordinated enzymatic and scaffolding activities. The mechanistic diversity of A20’s inhibitory actions underscores its therapeutic potential in modulating NF-κB, cell death, IL-17R, IL-1R, TLR and JAK/STAT signaling cascades implicated in chronic inflammatory disorders.

## 4 Genetic mouse models of A20 associated with psoriatic skin inflammation and arthritis

### 4.1 A20 haploinsufficiency and systemic inflammation

Genetic manipulation of A20 has enabled the development of animal models to study PsA and related inflammatory disorders. Complete A20 deficiency (A20-/-) in mice results in severe multi-organ inflammation, cachexia, and destructive arthritis, culminating in neonatal lethality within two weeks due to hypersensitivity to lipopolysaccharide (LPS) and TNF (47). In contrast, A20+/- heterozygous mice survive without overt health issues but exhibit heightened susceptibility to inflammatory stimuli. Studies demonstrate that A20+/- mice display exacerbated psoriasis-like skin inflammation upon imiquimod (IMQ) challenge, highlighting the role of A20 haploinsufficiency in amplifying inflammatory responses (36, 71).

### 4.2 Domain-specific mutations result in distinct phenotypes

To dissect the functional contributions of specific A20 domains, researchers have generated mice with mutations in different key motifs, such as OTU (C103A), ZnF4 (C609A, C612A), and ZnF7 (C764A, C767A). Mice with OTU or ZnF4 mutations exhibit increased sensitivity to TNF and colitis but do not develop spontaneous inflammation (41). In contrast, ZnF7 mutations result in PsA-like phenotypes, including psoriatic skin lesions, enthesitis, dactylitis, nail destruction, and distal interphalangeal joint (DIP) deformities. Notably, mice with combined ZnF4 and ZnF7 mutations display severe perinatal lethality with multi-organ inflammation, underscoring the synergistic roles of these domains in regulating inflammation (39).

### 4.3 Cell type-specific knockouts highlight the tissue-specific role of A20 in PsA pathogenesis

Cell type-specific A20 knockout models have further elucidated the tissue-specific functions of A20. Matmati, M. et al., generated a myeloid-specific A20 knockout mouse (A20^myel-KO^), which, unlike A20-/- mice, did not develop cachexia or die prematurely, but manifested destructive arthritis (72). A20^myel-KO^ mice were observed to develop paw swelling and redness at 8–12 weeks of age, and 100% of them developed arthritis by 20 weeks of age. Histological analysis and PET-CT revealed significant synovial and periarticular inflammation, as well as extensive cartilage destruction, bone destruction, and PIP deformations (72). Notably, joint inflammation of A20^myel-KO^ mice started at the posterior part of their ankles, and spread to the tarsal joints and tibiotalar joints. The inflammatory phenotypes of A20^myel-KO^ mice were limited to the joints. No signs of inflammation were found in other organs such as the skin, liver, intestines or lungs.

Declercq W, et al., found epidermis-specific A20 knockout (A20EKO) mice do not develop spontaneous skin lesions but exhibit keratinocyte hyperproliferation (73). These mice showed upregulated expression of IL-17 and TNF-α signaling-related cytokines and chemokines (e.g., TNF, Ccl20, Cxcl1, IL-22, IL-23a) and are more susceptible to psoriasis upon induction (74, 75). Although Declercq W et al. reported the absence of arthritis and immune cell infiltration in their model, Tobias, Ryan et al. found that mice with A20 deficiency in keratinocytes (A20^KIKO^) developed PsA-like manifestations (74, 76). These mice presented with spontaneous skin lesions, spontaneous nail lesions, DIP deformations, polyarticular arthritis, axial arthritis, enthesitis, bone erosion, new bone formation at their distal phalanxes, resembling PsA phenotypes observed in ZnF7-mutant mice. Unlike A20^myel-KO^ mice, whose inflammation began at the posterior part of the ankles, A20^KIKO^ mice first displayed inflammation at their distal phalanxes. Furthermore, the deformation of interphalangeal joints appeared in PIPs of A20^myel-KO^ mice, but DIPs of A20^KIKO^ and A20^mZnF7/mZnF7^ mice (76). This may explain the clinical heterogeneity that both DIP and PIP inflammation can occur in PsA patients, indicating deficient keratinocytes and myeloid cells contribute to different disease phenotypes (41).

However, in the dendritic cell (DC)-specific A20 knockout (CD11c-Cre A20fl/fl mice, the situation differs. 5~10% CD11c-Cre A20^fl/fl^ mice developed spontaneous skin lesions, and about 10% of them developed acute arthritis spontaneously at 4–6 months of age, with features of paw swelling, severe synovitis, and ehthesitis (77, 78). The acute arthritis subsides a few weeks after its onset, then became chronic with persistent enthesitis, joint ankylosis, cartilage and bone erosion, new bone formation and chondrogenesis in the limbs and spine for 1 year (77, 78). These mice are also more susceptible to IMQ-induced psoriatic skin inflammation. Given the low incidence of arthritis, it is thought that DCs may play a subsidiary role in causing PsA-like symptoms. Collectively, these models underscore the multifaceted role of A20 in regulating tissue-specific inflammatory responses and provide valuable tools for studying PsA.

In summary, A20-deficient mouse models have significantly advanced our understanding of PsA pathogenesis. **Table 1** summarizes the relevant models described above. By recapitulating key features of PsA, such as skin lesions, enthesitis, and joint inflammation, these models highlight the critical role of A20 in maintaining immune homeostasis and offer insights into the cellular and molecular mechanisms underlying PsA. Future research leveraging these models may pave the way for novel therapeutic strategies targeting A20 and its associated pathways.

**Table 1:** A20-deficient genetic mouse models for PsA.

Pathological features	A20+/–	A20^KIKO^ (A20^EKO)^	A20^ZF7/ZF7^	A20^myel-KO^	A20f^l/fl^ Cd11c-Cre
Spontaneous peripheral arthritis	–	✓	✓	✓	✓
Spontaneous axial arthritis	–	✓	No evidence		✓
Enthesitis	–	✓	✓	✓	✓
Synovitis	–	✓		✓	✓
Dactylitis	–	✓	✓	✓	–
DIP deformation	–	✓	✓	–	–
PIP deformation	–	–	–	✓	✓
Nail lesion	–	✓	✓	–	–
Bone erosion	–	✓	✓	✓	✓
New bone formation	–	✓	✓	✓	✓
spontaneous psoriatic skin lesions	–	–	✓	–	–
Susceptible to IMQ-induced psoriatic skin lesions	✓	✓	No evidence	No evidence	✓
IBD	–	Undetermined	Mild inflammation	–	√ (100%)
Serum antibodies	–	Undetermined	RF-, ANA↑, CCP↑	type II collagen↑	- (RF, ANA, CCP)

↑, upregulated.

## 5 The function of A20 in psoriatic skin and joint inflammation

### 5.1 Cytokine networks in PsA pathogenesis

**Figure 6:**
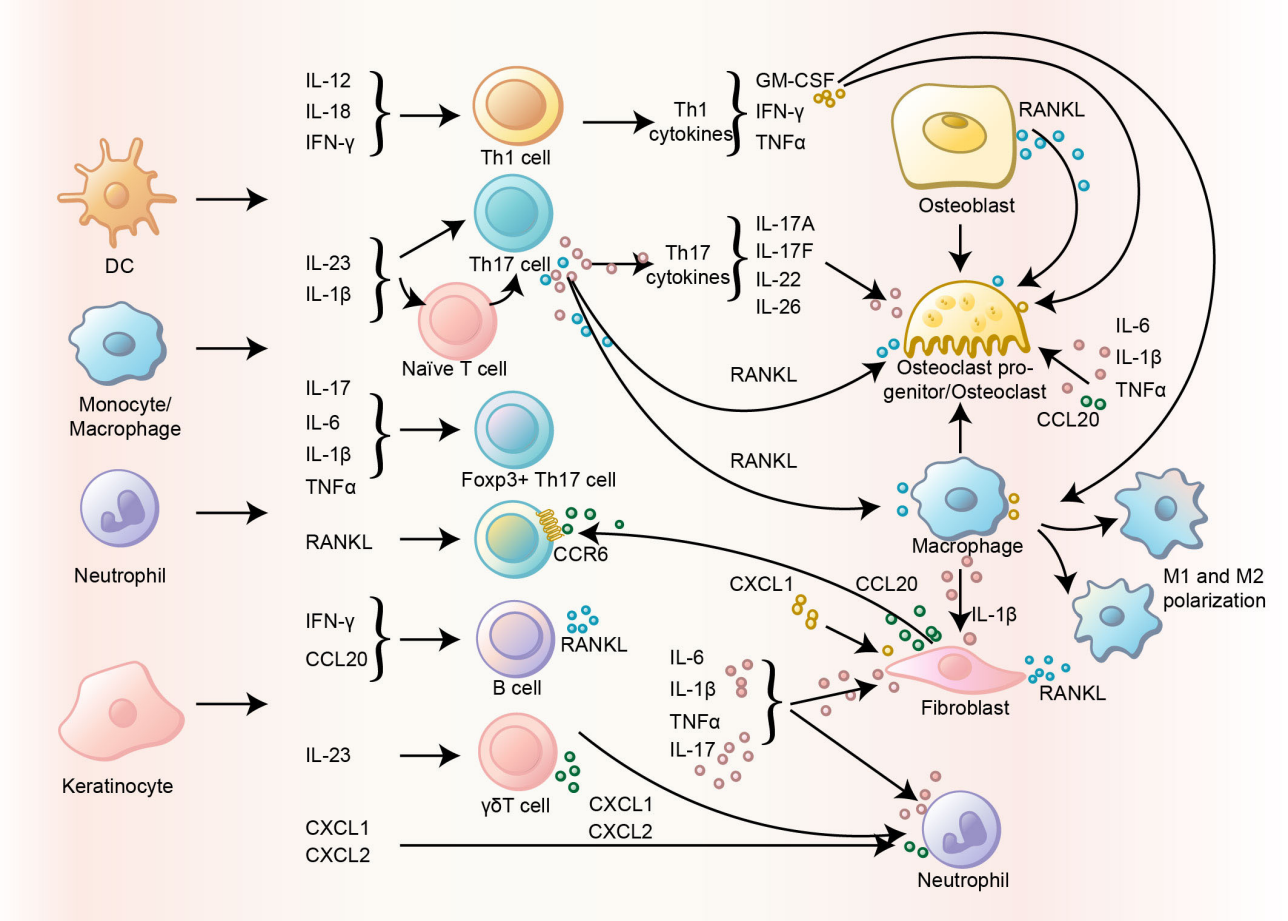
Pathological cytokines in PsA. Different cytokines slide between different immune and non-immune cells, and drives pathology of PsA.

A20 deficiency is closely associated with dysregulation of critical cytokines involved in psoriasis and PsA, including TNF, IL-6, IL-17, IL-23, CCL20, and CXCL10 (79). These cytokines are produced by both immune cells (e.g., T cells, macrophages, dendritic cells) and non-immune cells (e.g., keratinocytes), highlighting the complex interplay between different cell types in driving inflammation (**Figure 6**). The roles of these cytokines vary across cell types and disease models, underscoring the need for targeted therapeutic approaches.

#### 5.1.1 The IL-23/IL-17 axis drives PsA pathogenesis

The IL-23/IL-17 axis constitutes a critical signaling cascade governing inflammatory processes in psoriasis and PsA, with IL-23 serving as the primary upstream regulator and IL-17 acting as the central effector cytokine (80). Inhibitors toward this axis (such as bimekizumab, guselkumab, Secukinumab and ixekizumab), has achieved remarkable clinical efficacy (81).IL-23 initiates the pathogenic cascade by promoting the differentiation and expansion of IL-17-producing immune cells, particularly CD4+ T cells and γδT cells. While γδT cells producing IL-17 remain scarce in healthy individuals, they exhibit marked expansion in inflammatory milieus (82, 83). Notably, CD4-CD8-γδ+ IL-17-secreting γδT cells have been identified as essential mediators of enthesitis in PsA models (84, 85). Experimental evidence from A20+/– mice reveals systemic immune dysregulation characterized by elevated γδT cells, Gr-1-CD11b+ macrophages, and Gr-1+CD11b+ neutrophils in lymph nodes, mirroring immune alterations observed in human disease (71).

In A20-deficient murine models of imiquimod-induced inflammation, IL-23 orchestrates the recruitment of IL-17-producing γδT and CD4+ T cells to affected tissues through mechanisms modulated by the A20 protein (71). Molecular profiling demonstrates concurrent upregulation of IL-17A and IL-12/23p40 in cutaneous, lymphatic, and splenic tissues, paralleling findings in psoriasis patients with A20 haploinsufficiency (71). Therapeutic interventions reveal distinct roles within this axis: Complete resolution of ear swelling achieved through IL-12/23p40 neutralization contrasts with partial efficacy of IL-17 blockade, underscoring IL-23’s dominant regulatory position (71). In PsA models, IL-17A inhibition effectively prevents arthritis development, consistent with clinical responses observed in PsA therapeutics (39). The disproportionate elevation of serum IL-17 compared to synovial fluid levels suggests systemic rather than localized immune activation in PsA pathogenesis (86). Current evidence positions IL-17A as executing downstream inflammatory effects under IL-23 regulation, though precise mechanistic contributions to entheseal inflammation and bone remodeling require further elucidation. Emerging data from ZF7 murine models reinforce the therapeutic potential of IL-17 pathway modulation while highlighting residual disease components potentially mediated through alternative cytokine networks (39). These findings collectively establish the IL-23/IL-17 axis as a hierarchical signaling framework where IL-23 directs immune cell polarization and tissue homing, with IL-17 mediating subsequent effector responses in both cutaneous and articular manifestations of psoriasiform disease.

#### 5.1.2 The triad of TNF-α, IL-6, and IL-1β plays a critical role in PsA

Unlike IL-17A, TNF is considered more relevant to arthritis than skin inflammation (87). Studies using A20-deficient murine models highlight TNF’s critical involvement (39). For instance, A20^myel-KO^ mice exhibit destructive polyarthritis accompanied by elevated systemic levels of TNF, IL-1β, IL-6, and MCP-1, alongside localized joint increases in TNF, IL-1β, IL-6, and IL-23 (72). Peritoneal macrophages from these mice constitutively secrete TNF and IL-6, both implicated in osteoclastogenesis (36, 72). Intriguingly, while IL-6 neutralization ameliorates arthritis, TNF blockade shows inconsistent therapeutic efficacy (72). To clarify TNF’s indispensability, TNF-deficient A20^ZF7/ZF7^ mice were generated. Homozygous TNF deletion completely abolished arthritis development, whereas heterozygous deficiency prevented disease onset in most cases (39). These findings underscore TNF’s non-redundant role in arthritis pathogenesis, aligning with the clinical success of anti-TNF biologics in managing inflammatory arthropathies.

The synergistic interplay of TNF-α, IL-6, and IL-1β establishes a self-amplifying inflammatory loop central to arthritis progression. A2 is upregulated as a feedback mechanism to attenuate this cycle (88). Clinically, IL-6 is markedly elevated in the serum and synovial fluid of PsA patients, positioning it as a potential biomarker (89). However, therapeutic targeting of IL-6 with clazakizumab demonstrated limited efficacy in improving enthesitis, dactylitis, or joint inflammation, with no clear dose-response relationship, leading to discontinuation of clinical trials (90). IL-1β, another key mediator, is significantly upregulated in PsA patients and experimental models, where it exacerbates synovitis and bone erosion (91). Although Anakinra, an IL-1 receptor antagonist, has received regulatory approval for rheumatoid arthritis treatment, therapeutic targeting of IL-1β in psoriasis and PsA reveals heterogeneous clinical responses, suggesting context-dependent roles (92, 93). Collectively, these cytokines form an interdependent triad driving articular inflammation, yet their therapeutic targeting requires nuanced approaches to balance efficacy against complex immune redundancies.

#### 5.1.3 Chemokines mediate immune cell recruitment and drive the development of PsA

Except for the classic cytokines discussed above, chemokines were also found critical in the development of PsA. Compared to RA and OA, cytokines such as CXCL10 and CCL20 (which can be induced by A20 deficiency), but not IL-17 and IL-23, are assumed PsA characteristic according to clinical studies (86, 94, 95). CCL20, primarily synthesized by epithelial cells including keratinocytes and intestinal cells, undergoes transcriptional regulation through IL-17 signaling while being modulated by MyD88 and TNF-α pathways (74, 96, 97). This chemokine specifically interacts with CCR6, a receptor expressed on Th17 cells, regulatory T cells (Tregs), macrophages, dendritic cell precursors, and innate lymphoid cells (ILCs) (98). The CCL20-CCR6 axis has been demonstrated crucial in the enthesitis of IL-23 minicircle DNA-induced murine PsA model (99). Furthermore, the synovial fluid of PsA patients exhibits higher CCL20 concentrations than serum, implying localized chemotactic activity that recruits CCR6+ immune cells to dermal and articular tissues, subsequently driving IL-17 production (86). CXCL10, an inflammatory chemokine predominantly secreted by monocytes and macrophages, engages CXCR3 receptors expressed on both immune and resident tissue cells (100). While elevated synovial CXCL10 levels position it as a potential PsA serum biomarker, clinical observations present conflicting temporal patterns – displaying transient increases followed by progressive declines in chronic phases (101–103). This chemokine’s dual presence in immune and structural cells suggests participation in both inflammatory infiltration and tissue remodeling processes (104). Emerging evidence implicates additional mediators including CCL2, CCL5, CXCL1, and S100A9, which are associated with A20 deficiency in experimental model, warranting further investigation into their synergistic or compensatory roles. The complex interplay between epithelial-derived chemokines and recruited immune effectors highlights potential therapeutic targets, though current understanding necessitates deeper exploration of spatiotemporal regulation and receptor-ligand dynamics in PsA microenvironments.

#### 5.1.4 Cytokines in bone remodeling and resorption

Aberrant bone resorption is a common pathological process in arthritis, where cytokines and chemokines play vital roles. Despite its rigid appearance, bone undergoes continuous remodeling mediated by a complex network of cytokines (105, 106). Pro-osteoclastogenic cytokines, including RANKL, TNF-α, IL-6, IL-1β, IL-17A, IL-23, IL-7, IL-8, IL-15, IL-11, IL-34, IL-21, CCL2, CXCL10, and CXCL12, drive osteoclast differentiation and bone resorption. Conversely, anti-osteoclastogenic cytokines such as IFNs, IL-4, IL-10, IL-3, IL-12, IL-27, and IL-33 counterbalance these processes to maintain skeletal homeostasis.

A20 deficiency disrupts this balance, predominantly upregulating pro-osteoclastogenic cytokines. TNF-α and CCL20 directly enhance osteoclastogenesis, while IL-17 and IL-1β indirectly promote bone resorption by elevating RANKL expression in osteoblasts and stromal cells (70, 107). Synergistic interactions between cytokines further amplify pathological responses: CXCL10 and RANKL mutually upregulate each other, exacerbating osteoclastogenesis in the presence of CD4+ T cells (104). Additionally, late-phase NF-κB response genes (e.g., Csf2, Il6, C3, Ccl5 and Mmp3), rather than the early phase genes, were found increased in A20-deficient cells and in the paws of pre-diseased A20^ZF7/ZF7^ mice (39). This is consistent with the fact that the arthritis-correlated genes are mainly tanscribed in the late phase.

### 5.2 Hyperactive immune responses in PsA: mechanistic insights from A20-deficient models

#### 5.2.1 Adaptive immunity

Adaptive immunity is essential in the development of PsA. A case-control study identifies an upregulated lymphocyte ratio (PLR) in patients with PsA compared to those with plaque psoriasis, implicating a role of lymphocytes in PsA (108). Although T and B cells were found unnecessary for the development of arthritis in A20^myel-KO^ mice, studies of A20^ZF7/ZF7^ mice tells different (72). In A20^ZF7/ZF7^ mice, T cells exhibit heightened activation and proliferation compared to wild-type counterparts, whereas B cells remain unaffected (39). Notably, lymphocyte-deficient A20^ZF7/ZF7^ Rag1^KO^ mice failed to develop arthritis, underscoring the indispensable role of adaptive immune cells (39). However, B cell deficiency in A20^ZF7/ZF7^ mice did not mitigate arthritis, indicating that T cells, but not B cells, are essential drivers of disease progression (39). Additionally, the lack of T cells protected mice with A20-deficient keratinocytes from PsA-like pathology (76). The contrasting outcomes across murine models underscore the complexity of immune interactions, reinforcing the need for targeted exploration of T cell-specific mechanisms in future therapeutic strategies.

#### 5.2.2 Innate immunity and MyD88-dependent signaling

The pathogenesis of arthritis in A20-deficient murine models is critically dependent on MyD88-mediated innate immune signaling. Experimental evidence demonstrates that peritoneal macrophages from A20^mye-KO^ mice and bone marrow-derived macrophages (BMDMs) from A20^ZF7/ZF7^ mice exhibit hyperresponsiveness to lipopolysaccharide (LPS) stimulation (39, 72). This pathological response primarily engages the MyD88 adaptor protein rather than the Toll/IL-1R resistance (TIR) domain–containing adapter-inducing IFN-β (TRIF)-dependent pathway, as genetic ablation of MyD88 in both A20^myel-KO^ and A20^ZF7/ZF7^ mice completely prevents arthritis development, while TRIF deletion fails to mitigate disease progression (39, 41, 72). The critical upstream receptors driving this process include TLR4 and IL-1R. Therapeutic TLR4 blockade effectively suppressed arthritis in A20^myel-KO^ mice (72). Complementary studies using A20^myel-KO^Il1r^KO^ double-knockout mice reveal near-complete disease resolution, confirming the non-redundant roles of both TLR4 and IL-1R signaling pathways in arthritis pathogenesis (109).

MyD88-dependent signaling exerts its arthritogenic effects through both myeloid cells and non-myeloid cells. Notably, further knockout of MyD88 in synovial fibroblasts, inhibits arthritis caused by A20 myeloid knockout (41). Polykratis et al. delineated a critical IL-1β/MyD88 axis in SF activation, where macrophage-derived IL-1β stimulates SFs to produce metalloproteinases and inflammatory cytokines through MyD88 signaling (41, 72). This cellular crosstalk establishes a self-perpetuating inflammatory loop in joint tissues. In A20^KIKO^ mice, additional loss of MyD88 in keratinocytes, but not germline disruption of IFN receptors, also prevented the development of PsA-like disease, underscoring the broad, tissue-wide role of MyD88 (76).

The innate signaling pathways are intersected with adaptive immune responses, which is critically exemplified in dendritic cell (DC)-specific A20-deficient murine models. A20 exerts dual regulatory control over both MyD88-dependent and -independent signaling cascades within DCs (110). Genetic ablation of A20 in CD11c-Cre A20^fl/fl^ mice induces spontaneous DC activation, driving pathogenic Th1 and Th17 cell differentiation that underlies systemic inflammation (77). Paradoxically, *in vitro* stimulation of DCs from PsA patients with pathogenic stimuli and TLR ligands demonstrates suppressed pro-inflammatory cytokine release, despite concurrent upregulation of intracellular A20, SOCS3, and antimicrobial pathway components (111). This apparent discrepancy may reflect A20’s dual functionality in acute versus chronic inflammation, where its sustained expression in persistent disease states could stabilize negative feedback mechanisms to counterbalance inflammatory signaling.

#### 5.2.3 Necroptosis and NLRP3 inflammasome activation

The pathogenesis of PsA in A20-deficient models underscores the critical interplay between necroptosis and NLRP3 inflammasome activation. As stated above, A20 is pivotal in regulating NF-κB and TNFR1 signaling pathways. Experimental studies utilizing A20^myel-KO^ mice reveal that neither NF-κB inhibition via IKK2 deletion nor TNFR1 ablation fully prevents arthritis, suggesting redundant inflammatory pathways, such as TLR3/TLR4-TRIF or IFN receptor signaling, may compensate to sustain disease progression (41, 72). This highlights the complexity of inflammatory cascades in A20 deficiency and the need to explore alternative mechanisms driving joint pathology.

Central to arthritis development in these models is RIPK1-RIPK3-MLKL-mediated necroptosis. Genetic ablation of Ripk3 or Mlkl, or inhibition of RIPK1 kinase activity, significantly attenuates arthritis severity in A20^myel-KO^ mice. Notably, while RIPK3 or MLKL deletion suppresses IL-1β and IL-1α release from A20-deficient macrophages, loss of RIPK1 catalytic function does not, implying divergent roles for RIPK1 in necroptosis regulation (41). Emerging evidence suggests RIPK1 may exert non-catalytic scaffolding functions to propagate inflammation (112).

Concurrent with necroptosis, NLRP3 inflammasome hyperactivation emerges as a key driver of arthritis (41). A20^myel-KO^ mice exhibit elevated NLRP3 inflammasome activity in BMDMs, with genetic deletion of Nlrp3, Casp1/11, or ASC markedly reducing disease severity (109). Mechanistically, A20 deficiency amplifies both priming and activation phases of inflammasome signaling (58, 113). During priming, unchecked NF-κB activation upregulates NLRP3 and proIL-1β expression. In the activation phase, A20 loss lowers the threshold for NLRP3 oligomerization, facilitating IL-1β maturation. Studies indicate that A20’s zinc finger motifs, particularly ZnF4 and ZnF7, synergistically suppress inflammasome assembly, while NLRP3 activation reciprocally recruits A20 to inflammasomes, forming a negative feedback loop (39). Despite these regulatory mechanisms, inflammasome inhibition only partially alleviates arthritis, underscoring the contribution of inflammasome-independent pathways, including necroptosis, to disease pathogenesis.

**Figure 7:**
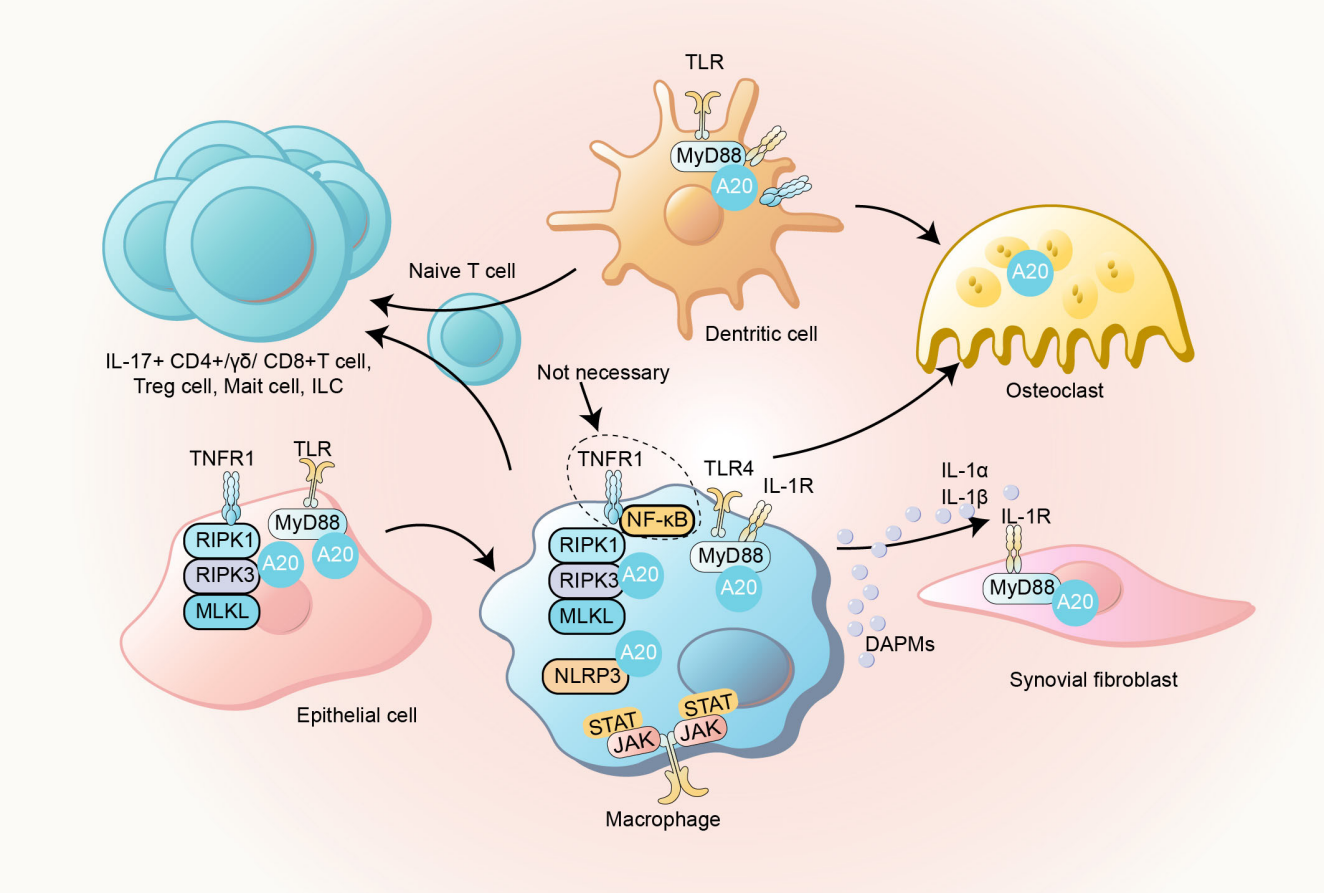
Communications between immune cells and non-immune cells. This schematic illustrates bidirectional interactions between immune and non-immune cells in PsA inflammatory processes. Macrophage necroptosis and NLRP3 inflammasome activation promote the release of DAMPs and IL-1β, which induce immune responses in synovial fibroblasts, osteoblasts, and osteoclasts. Non-immune cells, including keratinocytes and intestinal epithelial cells, exhibit immunomodulatory functions by driving inflammatory cascades. Concurrently, immune cells such as macrophages and dendritic cells amplify pathogenic Th17 cell responses, perpetuating inflammation in psoriatic skin lesions and arthritic joints.

The interplay between necroptosis and inflammasome activation in A20-deficient macrophages reveals a hierarchical relationship, with necroptosis predominating in driving tissue damage (41) (**Figure 7**). While A20’s ubiquitin-modifying functions are implicated in regulating both pathways, precise molecular mechanisms—such as its targeting of receptor complexes or inflammasome components—remain elusive (41). Intriguingly, RIPK3 and MLKL deletions not only block necroptosis but also reduce IL-1β levels, suggesting crosstalk between these pathways (41). This synergy may involve RIPK3-mediated NLRP3 activation or shared upstream regulators, though further studies are needed to delineate these interactions. As a consequence of the activation of cell death pathways, the released IL-1β subsequently stimulates synovial fibroblasts via IL-1R signaling, perpetuating synovitis.

In summary, A20 deficiency in PsA models unmasks a dual pathogenic axis: RIPK1-RIPK3-MLKL-dependent necroptosis and NLRP3 inflammasome hyperactivation. These pathways operate both independently and synergistically, with necroptosis serving as the primary driver of tissue injury and inflammasome activation amplifying IL-1β-mediated inflammation. Therapeutic strategies targeting both mechanisms—such as combined necroptosis inhibitors and NLRP3 antagonists—may offer enhanced efficacy in mitigating arthritis progression. However, unresolved questions persist regarding cell-type-specific contributions, the role of non-catalytic RIPK1 functions, and the temporal regulation of these pathways during disease evolution. Addressing these gaps will refine our understanding of PsA pathogenesis and guide precision therapeutic development.

#### 5.2.4 Regulation of osteoclast differentiation

Bone loss represents a critical pathological manifestation in PsA, occurring both systemically and locally. Systemic osteoporosis in PsA arises from excessive osteoclast activity, which disrupts bone remodeling homeostasis. Localized osteoclast hyperactivity contributes to periarticular bone erosion and irreversible joint deformities. A20 deficiency has been identified as a pivotal driver of accelerated osteoclastogenesis and associated osteoporosis. Experimental evidence from A20^mye-KO^ murine models demonstrates severe bone loss accompanied by elevated osteoclast-specific biomarkers and expanded populations of CD11b+Gr-1+ myeloid-derived suppressor cells (MDSCs) and CD115+CD117+ osteoclast precursors in splenic tissues (72).

*In vitro* analyses reveal enhanced osteoclast differentiation capacity in A20-deficient hematopoietic cells, with bone marrow-derived and peripheral blood leukocytes from A20^mye-KO^ mice forming osteoclasts exhibiting increased cell size, multinucleation frequency, and calcium phosphate resorptive activity compared to wild-type counterparts (36, 72). Cell-specific deletion studies further clarify A20’s direct regulatory role: osteoclast-restricted A20 knockout mice develop significant trabecular and cortical bone deterioration without systemic inflammation or arthritis, underscoring A20’s cell-autonomous function in bone metabolism (36).

Mechanistic investigations indicate A20 modulates osteoclast differentiation through multifaceted pathways, including suppression of necroptotic cell death, inhibition of Toll-like receptor (TLR) signaling cascades, and negative regulation of NF-κB activation (88, 114). Additionally, A20 exerts indirect control over osteoclastogenesis by modulating pro-inflammatory cytokine networks, particularly through downregulation of TNF-α, IL-1β, and IL-6 production, as stated above. These dual regulatory mechanisms position A20 as a critical molecular checkpoint in balancing bone resorption and formation, with its dysfunction contributing to pathological bone destruction in inflammatory arthropathies.

#### 5.2.5 JAK/STAT signaling

The JAK/STAT signaling pathway, an evolutionarily conserved cascade activated by cytokines, plays a critical role in immune regulation and has been implicated in the pathogenesis of A20 deficiency-related disorders (115, 116). Beyond its classical association with interferon (IFN) signaling, emerging evidence highlights its interaction with interleukin IL-17 and TNF signaling pathways (117). Clinically, inhibitors targeting this pathway, such as tofacitinib and upadacitinib, have demonstrated efficacy in managing psoriasis, PsA, and other autoimmune conditions, underscoring its therapeutic relevance (118–120).

Studies utilizing the A20^mye-KO^ murine model have elucidated the mechanistic link between myeloid-specific A20 deficiency and enthesitis (70). Myeloid A20 deficiency disrupts JAK/STAT signaling, leading to STAT1-driven inflammatory responses at entheseal sites (70). Notably, while prior research identified interactions between A20 and STAT3, the A20^mye-KO^ model revealed STAT1, rather than STAT3, as the primary mediator of enthesitis (69). Furthermore, the administration of tofacitinib, a jak inhibitor, alleviated enthesitis in mice (69). This divergence implicates the cell type-specific regulation of STAT proteins in inflammatory processes.

## 6 Discussion

A20 serves as a central regulator in PsA pathogenesis by mediating crosstalk between TNF and IL-17 signaling pathways, a mechanism that critically influences both disease dynamics and therapeutic efficacy (120). Genetic polymorphisms in TNFAIP3, such as rs2230926 and rs610604, correlate with enhanced responsiveness to TNF inhibitors, positioning A20 as a predictive biomarker for treatment stratification (12, 121). While anti-TNF therapy suppresses inflammation, it leads to elevated IL-17A levels and disease relapse in subsets of patients (122, 123). This can be explained by the disruption of the A20-mediated feedback loop. Mechanistically, TNF-α signaling via TNFR2 induces A20 expression, which in turn dampens IL-17A production by regulating p38 MAPK, PKC, JAK, and TCR pathways (124, 125). The therapeutic reduction of TNF-α consequently diminishes A20 levels, impairing its capacity to suppress IL-17A and creating a permissive environment for IL-17-driven inflammation (64, 124, 125). The therapeutic paradox underscores the necessity for precision medicine approaches incorporating A20 functional status assessment, particularly combination therapies merging TNF inhibition with IL-17 blockade in patients carrying TNFAIP3 risk variants. Preclinical validation using A20-deficient murine models demonstrates faithful recapitulation of PsA clinical manifestations – including arthritis, enthesitis, synovitis, and characteristic dermatological presentations – providing robust platforms for mechanistic exploration and targeted therapeutic development. These findings collectively position A20 as both a molecular linchpin in PsA pathophysiology and a stratification biomarker for optimizing biologic therapy selection.

Non-immune cells are integral to PsA pathogenesis through A20-dependent mechanisms. Studies in bone marrow chimeras reveal that A20 haploinsufficiency in non-hematopoietic cells exacerbates dermatitis, albeit less severely than systemic A20 deficiency, implicating both immune and stromal compartments in disease initiation (71). Reduced A20 expression in both lesional and non-lesional skin of PsA patients suggests that baseline A20 insufficiency, compounded by environmental triggers, primes tissues for inflammation (71). Experimental models using A20^myel-KO^ mice reveal synovial fibroblasts as pivotal mediators of arthritic pathology, with genetic ablation of Myd88 in these cells significantly attenuating disease progression (41). Complementary evidence from epidermis-specific A20 knockout mice demonstrates keratinocyte hyperproliferation and systemic inflammation characterized by elevated cutaneous cytokine/chemokine profiles recapitulating early psoriatic lesions (73, 74). Researchers also found that targeted epidermal A20 deletion induces spontaneous PsA-like manifestations, including periarticular inflammation, supporting the paradigm of skin-derived inflammatory mediators initiating joint pathology through chemotactic or direct cellular communication (74, 76). Elucidating the cellular communication between immune cells and non-immune cell involved in the pathological processes of the skin and joints may advance the development of novel therapeutic strategies (**Figure 7**).

Early diagnosis of PsA is hindered by nonspecific symptoms and a lack of validated biomarkers, but A20-deficient models offer transformative insights. Serum biomarkers such as IL-6 and CCL20, elevated in preclinical models, correlate with subclinical enthesitis and may predict articular progression in psoriasis patients (12). Advanced imaging techniques, including ultrasound and MRI, enhance detection of entheseal microdamage and axial involvement (axPsA), which are recapitulated in A20-deficient mice and often precede radiographic changes. These tools not only improve diagnostic accuracy but also enable monitoring of therapeutic responses, particularly for biologics targeting IL-23/IL-17 pathways. While osteoproliferation is also present in these models, its underlying mechanisms remain unexplored. Considering its significance in the diagnosis and prognosis of PsA patients, further research should be done in future studies. Furthermore, nail lesions—a clinical hallmark of PsA—show distinct molecular signatures in A20 models, suggesting their utility as early diagnostic indicators when combined with cytokine profiling.

In conclusion, A20 sits at the nexus of PsA’s molecular complexity, bridging immune dysregulation, stromal activation, and cell death pathways. Experimental models with A20 deficiency continue to provide critical insights into disease mechanisms and serve as essential platforms for evaluating targeted therapeutic strategies. Future research could prioritize spatiotemporal mapping of A20 activity across tissues, development of cell-specific delivery systems (e.g., entheseal-targeted nanoparticles). By addressing these challenges, the field can transition from reactive symptom management to mechanistically driven precision medicine, ultimately transforming PsA into a preventable or curable disease.

## References

[1] Griffiths CEM Armstrong AW Gudjonsson JE Barker JNWN . Psoriasis. Lancet. (2021) 397:1301–15. doi: 10.1016/S0140-6736(20)32549-6, PMID: 33812489

[2] Zhou X Chen Y Cui L Shi Y Guo C . Advances in the pathogenesis of psoriasis: from keratinocyte perspective. Cell Death Dis. (2022) 13:81. doi: 10.1038/s41419-022-04523-3, PMID: 35075118 PMC8786887

[3] Zalesak M Danisovic L Harsanyi S . Psoriasis and psoriatic arthritis-associated genes, cytokines, and human leukocyte antigens. Med (Kaunas). (2024) 60:815. doi: 10.3390/medicina60050815, PMID: 38792999 PMC11123327

[4] FitzGerald O Ogdie A Chandran V Coates LC Kavanaugh A Tillett W . Psoriatic arthritis. Nat Rev Dis Primers. (2021) 7:59. doi: 10.1038/s41572-021-00293-y, PMID: 34385474

[5] Tian Y Wu B Peng L Wang J Shen M . Three chinese pedigrees of A20 haploinsufficiency: clinical, cytokine and molecular characterization. Front Immunol. (2022) 13:955079. doi: 10.3389/fimmu.2022.955079, PMID: 35958611 PMC9360992

[6] Kadowaki T Kadowaki S Ohnishi H . A20 haploinsufficiency in east asia. Front Immunol. (2021) 12:780689. doi: 10.3389/fimmu.2021.780689, PMID: 34899744 PMC8664410

[7] Bowes J Barton A . The genetics of psoriatic arthritis: lessons from genome-wide association studies. Discov Med. (2010) 10:177–83., PMID: 20875338

[8] Momose M Hirota T Kikuchi S Inoue N Umezawa Y Nakagawa H . Associations of tnfaip3 variants with susceptibility to psoriasis vulgaris and psoriasis arthritis in a Japanese population. J Dermatol Sci. (2020) 100:220–2. doi: 10.1016/j.jdermsci.2020.09.005, PMID: 32998836

[9] Stuart PE Nair RP Tsoi LC Tejasvi T Das S Kang HM . Genome-wide association analysis of psoriatic arthritis and cutaneous psoriasis reveals differences in their genetic architecture. Am J Hum Genet. (2015) 97:816–36. doi: 10.1016/j.ajhg.2015.10.019, PMID: 26626624 PMC4678416

[10] Garrido AN Machhar R Cruz-Correa OF Ganatra D Crome SQ Wither J . Single-cell rna sequencing of circulating immune cells supports inhibition of tnfaip3 and nfkbia translation as psoriatic arthritis biomarkers. Front Immunol. (2025) 16:1483393. doi: 10.3389/fimmu.2025.1483393, PMID: 39991156 PMC11842318

[11] Curry PDK Morris AP Barton A Bluett J . Do genetics contribute to tnf inhibitor response prediction in psoriatic arthritis? Pharmacogenomics J. (2023) 23:1–7. doi: 10.1038/s41397-022-00290-8, PMID: 36243888 PMC9925377

[12] Tejasvi T Stuart PE Chandran V Voorhees JJ Gladman DD Rahman P . Tnfaip3 gene polymorphisms are associated with response to tnf blockade in psoriasis. J Invest Dermatol. (2012) 132:593–600. doi: 10.1038/jid.2011.376, PMID: 22113471 PMC3278539

[13] Radu A-F Bungau SG . Management of rheumatoid arthritis: an overview. Cells. (2021) 10:2857. doi: 10.3390/cells10112857, PMID: 34831081 PMC8616326

[14] Coates LC Soriano ER Corp N Bertheussen H Duffin KC Campanholo CB . Group for research and assessment of psoriasis and psoriatic arthritis (Grappa): updated treatment recommendations for psoriatic arthritis 2021. Nat Rev Rheumatol. (2022) 18:465–79. doi: 10.1038/s41584-022-00798-0, PMID: 35761070 PMC9244095

[15] Kaushik SB Lebwohl MG . Psoriasis: which therapy for which patient: psoriasis comorbidities and preferred systemic agents. J Am Acad Dermatol. (2019) 80:27–40. doi: 10.1016/j.jaad.2018.06.057, PMID: 30017705

[16] Fassio A Giovannini I Idolazzi L Zabotti A Iagnocco A Sakellariou G . Nail ultrasonography for psoriatic arthritis and psoriasis patients: A systematic literature review. Clin Rheumatol. (2020) 39:1391–404. doi: 10.1007/s10067-019-04748-2, PMID: 31440917

[17] Leung YY Ogdie A Orbai AM Tillett W Coates LC Strand V . Classification and outcome measures for psoriatic arthritis. Front Med (Lausanne). (2018) 5:246. doi: 10.3389/fmed.2018.00246, PMID: 30238006 PMC6135872

[18] Taylor W Gladman D Helliwell P Marchesoni A Mease P Mielants H . Classification criteria for psoriatic arthritis: development of new criteria from a large international study. Arthritis Rheum. (2006) 54:2665–73. doi: 10.1002/art.21972, PMID: 16871531

[19] Polachek A Furer V Zureik M Nevo S Mendel L Levartovsky D . Ultrasound, magnetic resonance imaging and radiography of the finger joints in psoriatic arthritis patients. Rheumatol (Oxford). (2022) 61:563–71. doi: 10.1093/rheumatology/keab272, PMID: 33734348

[20] Żelnio E Taljanovic M Mańczak M Sudoł-Szopińska I . Hand and wrist involvement in seropositive rheumatoid arthritis, seronegative rheumatoid arthritis, and psoriatic arthritis-the value of classic radiography. J Clin Med. (2023) 12:2622. doi: 10.3390/jcm12072622, PMID: 37048705 PMC10095289

[21] Sakellariou G Girolimetto N Tinazzi I Canzoni M Filippou G Batticciotto A . The ultrasonographic spectrum of toe dactylitis in psoriatic arthritis: A descriptive analysis. Clin Rheumatol. (2025) 44:1939–47. doi: 10.1007/s10067-025-07395-y, PMID: 40111541 PMC12078354

[22] Lisco G De Tullio A Zupo R Prete M Piazzolla G Racanelli V . Metabolic-associated enthesitis: A review on pathophysiology, clinical relevance, diagnostic challenges, and perspective on target treatments. Immunol Res. (2025) 73:106. doi: 10.1007/s12026-025-09655-0, PMID: 40646307

[23] Hammer GE Ma A . Molecular control of steady-state dendritic cell maturation and immune homeostasis. Annu Rev Immunol. (2013) 31:743–91. doi: 10.1146/annurev-immunol-020711-074929, PMID: 23330953 PMC4091962

[24] Cambré I Gaublomme D Schryvers N Lambrecht S Lories R Venken K . Running promotes chronicity of arthritis by local modulation of complement activators and impairing T regulatory feedback loops. Ann Rheum Dis. (2019) 78:787–95. doi: 10.1136/annrheumdis-2018-214627, PMID: 30928902

[25] Corte G Atzinger A Temiz SA Noversa de Sousa R Mutlu MY Schoenau V . Anatomical pattern of entheseal and synovial fibroblast activation in patients with psoriasis and its risk of developing psoriatic arthritis. RMD Open. (2024) 10:e004294. doi: 10.1136/rmdopen-2024-004294, PMID: 38862244 PMC11168197

[26] Zabotti A De Marco G Gossec L Baraliakos X Aletaha D Iagnocco A . Eular points to consider for the definition of clinical and imaging features suspicious for progression from psoriasis to psoriatic arthritis. Ann Rheumatic Dis. (2023) 82:1162–70. doi: 10.1136/ard-2023-224148, PMID: 37295926

[27] Stober C . Pathogenesis of psoriatic arthritis. Best Pract Res Clin Rheumatol. (2021) 35:101694. doi: 10.1016/j.berh.2021.101694, PMID: 34108102

[28] Ayan G Sadic A Kilic L Kalyoncu U . Degenerative and inflammatory osteoproliferations in lumbar radiographs in psoriatic arthritis patients. J Clin Med. (2022) 11:2009. doi: 10.3390/jcm11072009, PMID: 35407618 PMC9000142

[29] Hammitzsch A Ossadnik A Bachmann Q Merwald-Fraenk H Lorenz G Witt M . Increased interleukin-26 in the peripheral joints of patients with axial spondyloarthritis and psoriatic arthritis, co-localizing with cd68-positive synoviocytes. Front Immunol. (2024) 15:1355824. doi: 10.3389/fimmu.2024.1355824, PMID: 38799447 PMC11127564

[30] Cantini F Niccoli L Nannini C Cassarà E Kaloudi O Giulio Favalli E . Tailored first-line biologic therapy in patients with rheumatoid arthritis, spondyloarthritis, and psoriatic arthritis. Semin Arthritis Rheum. (2016) 45:519–32. doi: 10.1016/j.semarthrit.2015.10.001, PMID: 26607440

[31] Conigliaro P Chimenti MS Triggianese P Sunzini F Novelli L Perricone C . Autoantibodies in inflammatory arthritis. Autoimmun Rev. (2016) 15:673–83. doi: 10.1016/j.autrev.2016.03.003, PMID: 26970491

[32] Cockram PE Kist M Prakash S Chen S-H Wertz IE Vucic D . Ubiquitination in the regulation of inflammatory cell death and cancer. Cell Death Differentiation. (2021) 28:591–605. doi: 10.1038/s41418-020-00708-5, PMID: 33432113 PMC7798376

[33] Wu Y He X Huang N Yu J Shao B . A20: A master regulator of arthritis. Arthritis Res Ther. (2020) 22:220. doi: 10.1186/s13075-020-02281-1, PMID: 32958016 PMC7504854

[34] Shembade N Ma A Harhaj EW . Inhibition of nf-kappab signaling by A20 through disruption of ubiquitin enzyme complexes. Science. (2010) 327:1135–9. doi: 10.1126/science.1182364, PMID: 20185725 PMC3025292

[35] De A Dainichi T Rathinam CV Ghosh S . The deubiquitinase activity of A20 is dispensable for nf-κb signaling. EMBO Rep. (2014) 15:775–83. doi: 10.15252/embr.201338305, PMID: 24878851 PMC4196981

[36] Martens A Hertens P Priem D Rinotas V Meletakos T Gennadi M . A20 controls rank-dependent osteoclast formation and bone physiology. EMBO Rep. (2022) 23:e55233. doi: 10.15252/embr.202255233, PMID: 36194667 PMC9724664

[37] Barnabei L Laplantine E Mbongo W Rieux-Laucat F Weil R . Nf-κb: at the borders of autoimmunity and inflammation. Front Immunol. (2021) 12:716469. doi: 10.3389/fimmu.2021.716469, PMID: 34434197 PMC8381650

[38] Guo Q Jin Y Chen X Ye X Shen X Lin M . Nf-κb in biology and targeted therapy: new insights and translational implications. Signal Transduction Targeted Ther. (2024) 9:53. doi: 10.1038/s41392-024-01757-9, PMID: 38433280 PMC10910037

[39] Razani B Whang MI Kim FS Nakamura MC Sun X Advincula R . Non-catalytic ubiquitin binding by A20 prevents psoriatic arthritis–like disease and inflammation. Nat Immunol. (2020) 21:422–33. doi: 10.1038/s41590-020-0634-4, PMID: 32205880 PMC7195210

[40] Heyninck K De Valck D Vanden Berghe W Van Criekinge W Contreras R Fiers W . The zinc finger protein A20 inhibits tnf-induced nf-kappab-dependent gene expression by interfering with an rip- or traf2-mediated transactivation signal and directly binds to a novel nf-kappab-inhibiting protein abin. J Cell Biol. (1999) 145:1471–82. doi: 10.1083/jcb.145.7.1471, PMID: 10385526 PMC2133159

[41] Polykratis A Martens A Eren RO Shirasaki Y Yamagishi M Yamaguchi Y . A20 prevents inflammasome-dependent arthritis by inhibiting macrophage necroptosis through its znf7 ubiquitin-binding domain. Nat Cell Biol. (2019) 21:731–42. doi: 10.1038/s41556-019-0324-3, PMID: 31086261

[42] Zhang SQ Kovalenko A Cantarella G Wallach D . Recruitment of the ikk signalosome to the P55 tnf receptor: rip and A20 bind to nemo (Ikkgamma) upon receptor stimulation. Immunity. (2000) 12:301–11. doi: 10.1016/s1074-7613(00)80183-1, PMID: 10755617

[43] Yamaguchi N Oyama M Kozuka-Hata H Inoue J . Involvement of A20 in the molecular switch that activates the non-canonical nf-Кb pathway. Sci Rep. (2013) 3:2568. doi: 10.1038/srep02568, PMID: PMC3764444 24008839

[44] Bosanac I Wertz IE Pan B Yu C Kusam S Lam C . Ubiquitin binding to A20 znf4 is required for modulation of nf-κb signaling. Mol Cell. (2010) 40:548–57. doi: 10.1016/j.molcel.2010.10.009, PMID: 21095585

[45] Verhelst K Carpentier I Kreike M Meloni L Verstrepen L Kensche T . A20 inhibits lubac-mediated nf-κb activation by binding linear polyubiquitin chains via its zinc finger 7. EMBO J. (2012) 31:3845–55. doi: 10.1038/emboj.2012.240, PMID: 23032186 PMC3463847

[46] Skaug B Chen J Du F He J Ma A Chen ZJ . Direct, noncatalytic mechanism of ikk inhibition by A20. Mol Cell. (2011) 44:559–71. doi: 10.1016/j.molcel.2011.09.015, PMID: 22099304 PMC3237303

[47] Lee EG Boone DL Chai S Libby SL Chien M Lodolce JP . Failure to regulate tnf-induced nf-kappab and cell death responses in A20-deficient mice. Science. (2000) 289:2350–4. doi: 10.1126/science.289.5488.2350, PMID: 11009421 PMC3582399

[48] Song HY Rothe M Goeddel DV . The tumor necrosis factor-inducible zinc finger protein A20 interacts with traf1/traf2 and inhibits nf-kappab activation. Proc Natl Acad Sci United States America. (1996) 93:6721–5. doi: 10.1073/pnas.93.13.6721, PMID: 8692885 PMC39093

[49] Chawla M Mukherjee T Deka A Chatterjee B Sarkar UA Singh AK . An epithelial <I>Nfkb2</I> pathway exacerbates intestinal inflammation by supplementing latent rela dimers to the canonical nf-&X3ba;B module. Proc Natl Acad Sci. (2021) 118:e2024828118. doi: 10.1073/pnas.2024828118, PMID: 34155144 PMC8237674

[50] Draber P Kupka S Reichert M Draberova H Lafont E de Miguel D . Lubac-recruited cyld and A20 regulate gene activation and cell death by exerting opposing effects on linear ubiquitin in signaling complexes. Cell Rep. (2015) 13:2258–72. doi: 10.1016/j.celrep.2015.11.009, PMID: 26670046 PMC4688036

[51] Öztürk S Schleich K Lavrik IN . Cellular flice-like inhibitory proteins (C-flips): fine-tuners of life and death decisions. Exp Cell Res. (2012) 318:1324–31. doi: 10.1016/j.yexcr.2012.01.019, PMID: 22309778

[52] Zhang J Yang Y He W Sun L . Necrosome core machinery: mlkl. Cell Mol Life sciences: CMLS. (2016) 73:2153–63. doi: 10.1007/s00018-016-2190-5, PMID: 27048809 PMC11108342

[53] Oberst A Dillon CP Weinlich R McCormick LL Fitzgerald P Pop C . Catalytic activity of the caspase-8–flipl complex inhibits ripk3-dependent necrosis. Nature. (2011) 471:363–7. doi: 10.1038/nature09852, PMID: 21368763 PMC3077893

[54] Zhang X Fan C Zhang H Zhao Q Liu Y Xu C . Mlkl and fadd are critical for suppressing progressive lymphoproliferative disease and activating the nlrp3 inflammasome. Cell Rep. (2016) 16:3247–59. doi: 10.1016/j.celrep.2016.06.103, PMID: 27498868 PMC7191534

[55] Fritsch M Günther SD Schwarzer R Albert M-C Schorn F Werthenbach JP . Caspase-8 is the molecular switch for apoptosis, necroptosis and pyroptosis. Nature. (2019) 575:683–7. doi: 10.1038/s41586-019-1770-6, PMID: 31748744

[56] Günther C Martini E Wittkopf N Amann K Weigmann B Neumann H . Caspase-8 regulates tnf-α-induced epithelial necroptosis and terminal ileitis. Nature. (2011) 477:335–9. doi: 10.1038/nature10400, PMID: 21921917 PMC3373730

[57] Meng Y Sandow JJ Czabotar PE Murphy JM . The regulation of necroptosis by post-translational modifications. Cell Death Differentiation. (2021) 28:861–83. doi: 10.1038/s41418-020-00722-7, PMID: 33462412 PMC7937688

[58] Onizawa M Oshima S Schulze-Topphoff U Oses-Prieto JA Lu T Tavares R . The ubiquitin-modifying enzyme A20 restricts ubiquitination of the kinase ripk3 and protects cells from necroptosis. Nat Immunol. (2015) 16:618–27. doi: 10.1038/ni.3172, PMID: 25939025 PMC4439357

[59] Bai W Huo S Li J Shao J . Advances in the study of the ubiquitin-editing enzyme A20. Front Pharmacol. (2022) 13:845262. doi: 10.3389/fphar.2022.845262, PMID: 35592427 PMC9110840

[60] Priem D Devos M Druwé S Martens A Slowicka K Ting AT . A20 protects cells from tnf-induced apoptosis through linear ubiquitin-dependent and -independent mechanisms. Cell Death Dis. (2019) 10:692. doi: 10.1038/s41419-019-1937-y, PMID: 31534131 PMC6751190

[61] Li M Liu Y Xu C Zhao Q Liu J Xing M . Ubiquitin-binding domain in abin1 is critical for regulating cell death and inflammation during development. Cell Death Differentiation. (2022) 29:2034–45. doi: 10.1038/s41418-022-00994-1, PMID: 35430614 PMC9525631

[62] Liu Y Xu K Yao Y Liu Z . Current research into A20 mediation of allergic respiratory diseases and its potential usefulness as a therapeutic target. Front Immunol. (2023) 14:1166928. doi: 10.3389/fimmu.2023.1166928, PMID: 37056760 PMC10086152

[63] Zhong Z Su G Kijlstra A Yang P . Activation of the interleukin-23/interleukin-17 signaling pathway in autoinflammatory and autoimmune uveitis. Prog Retinal Eye Res. (2021) 80:100866. doi: 10.1016/j.preteyeres.2020.100866, PMID: 32422390

[64] Garg AV Ahmed M Vallejo AN Ma A Gaffen SL . The deubiquitinase A20 mediates feedback inhibition of interleukin-17 receptor signaling. Sci Signal. (2013) 6:ra44. doi: 10.1126/scisignal.2003699, PMID: 23737552 PMC4028484

[65] Amatya N Garg AV Gaffen SL . Il-17 signaling: the yin and the yang. Trends Immunol. (2017) 38:310–22. doi: 10.1016/j.it.2017.01.006, PMID: 28254169 PMC5411326

[66] Duan T Du Y Xing C Wang HYY Wang R-F . Toll-like receptor signaling and its role in cell-mediated immunity. Front Immunol. (2022) 13:812774. doi: 10.3389/fimmu.2022.812774, PMID: 35309296 PMC8927970

[67] Xue C Yao Q Gu X Shi Q Yuan X Chu Q . Evolving cognition of the jak-stat signaling pathway: autoimmune disorders and cancer. Signal Transduct Target Ther. (2023) 8:204. doi: 10.1038/s41392-023-01468-7, PMID: 37208335 PMC10196327

[68] Antonia RJ Karelehto E Toriguchi K Matli M Warren RS Pfeffer LM . Stat3 regulates inflammatory cytokine production downstream of tnfr1 by inducing expression of tnfaip3/A20. J Cell Mol Med. (2022) 26:4591–601. doi: 10.1111/jcmm.17489, PMID: 35841281 PMC9357623

[69] Liu X Mao Y Kang Y He L Zhu B Zhang W . Microrna-127 promotes anti-microbial host defense through restricting A20-mediated de-ubiquitination of stat3. iScience. (2020) 23:100763. doi: 10.1016/j.isci.2019.100763, PMID: 31958753 PMC6992901

[70] De Wilde K Martens A Lambrecht S Jacques P Drennan MB Debusschere K . A20 inhibition of stat1 expression in myeloid cells: A novel endogenous regulatory mechanism preventing development of enthesitis. Ann Rheum Dis. (2017) 76:585–92. doi: 10.1136/annrheumdis-2016-209454, PMID: 27551052

[71] Aki A Nagasaki M Malynn BA Ma A Kagari T . Hypomorphic A20 expression confers susceptibility to psoriasis. PloS One. (2017) 12:e0180481. doi: 10.1371/journal.pone.0180481, PMID: 28658319 PMC5489224

[72] Matmati M Jacques P Maelfait J Verheugen E Kool M Sze M . A20 (Tnfaip3) deficiency in myeloid cells triggers erosive polyarthritis resembling rheumatoid arthritis. Nat Genet. (2011) 43:908–12. doi: 10.1038/ng.874, PMID: 21841782

[73] Lippens S Lefebvre S Gilbert B Sze M Devos M Verhelst K . Keratinocyte-specific ablation of the nf-κb regulatory protein A20 (Tnfaip3) reveals a role in the control of epidermal homeostasis. Cell Death Differ. (2011) 18:1845–53. doi: 10.1038/cdd.2011.55, PMID: 21566665 PMC3214908

[74] Devos M Mogilenko DA Fleury S Gilbert B Becquart C Quemener S . Keratinocyte expression of A20/tnfaip3 controls skin inflammation associated with atopic dermatitis and psoriasis. J Invest Dermatol. (2019) 139:135–45. doi: 10.1016/j.jid.2018.06.191, PMID: 30118730

[75] Harirchian P Lee J Hilz S Sedgewick AJ Perez White BE Kesling MJ . A20 and abin1 suppression of a keratinocyte inflammatory program with a shared single-cell expression signature in diverse human rashes. J Invest Dermatol. (2019) 139:1264–73. doi: 10.1016/j.jid.2018.10.046, PMID: 30543901 PMC6642632

[76] TobiasR KumarS LiuJ LenciN GharabiA StiborD . Unrestrained myd88 signaling in A20-deficient keratinocytes triggers T-cell-dependent psoriatic arthritis-like disease. J Invest Dermatol. (2025) 145:2998–3010(e3). doi: 10.1016/j.jid.2025.03.043, PMID: 40316204

[77] Hammer GE Turer EE Taylor KE Fang CJ Advincula R Oshima S . Expression of A20 by dendritic cells preserves immune homeostasis and prevents colitis and spondyloarthritis. Nat Immunol. (2011) 12:1184–93. doi: 10.1038/ni.2135, PMID: 22019834 PMC3419270

[78] Kool M van Loo G Waelput W De Prijck S Muskens F Sze M . The ubiquitin-editing protein A20 prevents dendritic cell activation, recognition of apoptotic cells, and systemic autoimmunity. Immunity. (2011) 35:82–96. doi: 10.1016/j.immuni.2011.05.013, PMID: 21723156

[79] Li H Wang C Li X Kong Y Sun W . A20 deficiency in myeloid cells deteriorates the onset of vitiligo in mice. Dermatol Ther. (2021) 34:e14923. doi: 10.1111/dth.14923, PMID: 33651436

[80] Vecellio M Hake VX Davidson C Carena MC Wordsworth BP Selmi C . The il-17/il-23 axis and its genetic contribution to psoriatic arthritis. Front Immunol. (2021) 11:596086. doi: 10.3389/fimmu.2020.596086, PMID: 33574815 PMC7871349

[81] Kimball AB Jemec GBE Sayed CJ Kirby JS Prens E Ingram JR . Efficacy and safety of bimekizumab in patients with moderate-to-severe hidradenitis suppurativa (Be heard I and be heard ii): two 48-week, randomized, double-blind, placebo-controlled, multicenter phase 3 trials. Lancet. (2024) 403:2504–19. doi: 10.1016/s0140-6736(24)00101-6, PMID: 38795716

[82] Hu Y Hu Q Li Y Lu L Xiang Z Yin Z . Γδ T cells: origin and fate, subsets, diseases and immunotherapy. Signal Transduction Targeted Ther. (2023) 8:434. doi: 10.1038/s41392-023-01653-8, PMID: 37989744 PMC10663641

[83] Qi C Wang Y Li P Zhao J . Gamma delta T cells and their pathogenic role in psoriasis. Front Immunol. (2021) 12:627139. doi: 10.3389/fimmu.2021.627139, PMID: 33732249 PMC7959710

[84] Sherlock JP Joyce-Shaikh B Turner SP Chao C-C Sathe M Grein J . Il-23 induces spondyloarthropathy by acting on ror-Γt+ Cd3+Cd4–cd8– entheseal resident T cells. Nat Med. (2012) 18:1069–76. doi: 10.1038/nm.2817, PMID: 22772566

[85] Reinhardt A Yevsa T Worbs T Lienenklaus S Sandrock I Oberdörfer L . Interleukin-23–dependent Γ/Δ T cells produce interleukin-17 and accumulate in the enthesis, aortic valve, and ciliary body in mice. Arthritis Rheumatol. (2016) 68:2476–86. doi: 10.1002/art.39732, PMID: 27111864

[86] Mrabet D Laadhar L Sahli H Zouari B Haouet S Makni S . Synovial fluid and serum levels of il-17, il-23, and ccl-20 in rheumatoid arthritis and psoriatic arthritis: A Tunisian cross-sectional study. Rheumatol Int. (2013) 33:265–6. doi: 10.1007/s00296-011-2231-1, PMID: 22083617

[87] Jang D-i Lee AH Shin H-Y Song H-R Park J-H Kang T-B . The role of tumor necrosis factor alpha (Tnf-α) in autoimmune disease and current tnf-α Inhibitors in therapeutics. Int J Mol Sci. (2021) 22:1213448. doi: 10.3390/ijms22052719, PMID: 33800290 PMC7962638

[88] Kim SK Choe JY Park KY . Cpg oligodeoxynucleotides inhibit rankl-induced osteoclast formation by upregulating A20 deubiquitinase in raw 264.7 cells. Mediators Inflammation. (2022) 2022:5255935. doi: 10.1155/2022/5255935, PMID: 36091665 PMC9453122

[89] Choy EH De Benedetti F Takeuchi T Hashizume M John MR Kishimoto T . Translating il-6 biology into effective treatments. Nat Rev Rheumatol. (2020) 16:335–45. doi: 10.1038/s41584-020-0419-z, PMID: 32327746 PMC7178926

[90] Kaneko Y Takeuchi T . An update on the pathogenic role of il-6 in rheumatic diseases. Cytokine. (2021) 146:155645. doi: 10.1016/j.cyto.2021.155645, PMID: 34303949

[91] Tylutka A Walas L Zembron-Lacny A . Level of il-6, tnf, and il-1β and age-related diseases: A systematic review and meta-analysis. Front Immunol. (2024) 15:1330386. doi: 10.3389/fimmu.2024.1330386, PMID: 38495887 PMC10943692

[92] Acquitter M Plantin P Kupfer I Auvinet H Marhadour T . Anakinra improves pyoderma gangrenosum in psoriatic arthritis: A case report. Ann Intern Med. (2015) 163:70–1. doi: 10.7326/l15-5107, PMID: 26148290

[93] Jung N Hellmann M Hoheisel R Lehmann C Haase I Perniok A . An open-label pilot study of the efficacy and safety of anakinra in patients with psoriatic arthritis refractory to or intolerant of methotrexate (Mtx). Clin Rheumatol. (2010) 29:1169–73. doi: 10.1007/s10067-010-1504-5, PMID: 20532937

[94] Onuora S . Targeting the ccr6-ccl20 axis improves experimental psa. Nat Rev Rheumatol. (2021) 17:441–. doi: 10.1038/s41584-021-00663-6, PMID: 34226726

[95] Ruscitti P Esposito M Di Cola I Pellegrini C De Berardinis A Mastrangelo M . Cytokine profile characterization of naive patients with psoriasis and psoriatic arthritis: implications for a pathogenic disease continuum. Front Immunol. (2023) 14:1229516. doi: 10.3389/fimmu.2023.1229516, PMID: 37520537 PMC10373502

[96] Duan X Liu X Liu N Huang Y Jin Z Zhang S . Inhibition of keratinocyte necroptosis mediated by ripk1/ripk3/mlkl provides a protective effect against psoriatic inflammation. Cell Death Dis. (2020) 11:134. doi: 10.1038/s41419-020-2328-0, PMID: 32075957 PMC7031250

[97] Kadomoto S Izumi K Mizokami A . The ccl20-ccr6 axis in cancer progression. Int J Mol Sci. (2020) 21:5186. doi: 10.3390/ijms21155186, PMID: 32707869 PMC7432448

[98] Meitei HT Jadhav N Lal G . Ccr6-ccl20 axis as a therapeutic target for autoimmune diseases. Autoimmun Rev. (2021) 20:102846. doi: 10.1016/j.autrev.2021.102846, PMID: 33971346

[99] Shi Z Garcia-Melchor E Wu X Getschman AE Nguyen M Rowland DJ . Targeting the ccr6/ccl20 axis in entheseal and cutaneous inflammation. Arthritis Rheumatol. (2021) 73:2271–81. doi: 10.1002/art.41882, PMID: 34081845

[100] Pulito-Cueto V Armesto S Remuzgo-Martinez S Lopez-Mejias R Sebastian Mora-Gil M Ocejo-Vinyals JG . Characterizing the pre-disease state of psoriatic arthritis: cxcl10 levels are associated with subclinical synovitis in patients with psoriasis. Ann Rheumatic Dis. (2023) 82:1234–. doi: 10.1136/annrheumdis-2023-eular.5466

[101] Penkava F Velasco-Herrera MDC Young MD Yager N Nwosu LN Pratt AG . Single-cell sequencing reveals clonal expansions of pro-inflammatory synovial cd8 T cells expressing tissue-homing receptors in psoriatic arthritis. Nat Commun. (2020) 11:4767. doi: 10.1038/s41467-020-18513-6, PMID: 32958743 PMC7505844

[102] Barbarroja N López-Montilla MD Cuesta-López L Pérez-Sánchez C Ruiz-Ponce M López-Medina C . Characterization of the inflammatory proteome of synovial fluid from patients with psoriatic arthritis: potential treatment targets. Front Immunol. (2023) 14:1133435. doi: 10.3389/fimmu.2023.1133435, PMID: 37033920 PMC10073963

[103] Marzaioli V Canavan M Floudas A Flynn K Mullan R Veale DJ . Cd209/cd14(+) dendritic cells characterization in rheumatoid and psoriatic arthritis patients: activation, synovial infiltration, and therapeutic targeting. Front Immunol. (2021) 12:722349. doi: 10.3389/fimmu.2021.722349, PMID: 35095831 PMC8789658

[104] Lee EY Lee ZH Song YW . The interaction between cxcl10 and cytokines in chronic inflammatory arthritis. Autoimmun Rev. (2013) 12:554–7. doi: 10.1016/j.autrev.2012.10.001, PMID: 23092582

[105] Song JE Kim JS Shin JH Moon KW Park JK Park KS . Role of synovial exosomes in osteoclast differentiation in inflammatory arthritis. Cells. (2021) 10:120. doi: 10.3390/cells10010120, PMID: 33435236 PMC7827682

[106] Iwamoto N Kawakami A . The monocyte-to-osteoclast transition in rheumatoid arthritis: recent findings. Front Immunol. (2022) 13:998554. doi: 10.3389/fimmu.2022.998554, PMID: 36172385 PMC9510592

[107] Pandolfi F Franza L Carusi V Altamura S Andriollo G Nucera E . Interleukin-6 in rheumatoid arthritis. Int J Mol Sci. (2020) 21:5238. doi: 10.3390/ijms21155238, PMID: 32718086 PMC7432115

[108] Liu X Zhao Y Mu Z Jia Y Liu C Zhang J . The combination of il-6, plr and nail psoriasis: screen for the early diagnosis of psoriatic arthritis. Clin Cosmet Investig Dermatol. (2023) 16:1703–13. doi: 10.2147/ccid.S413853, PMID: 37404370 PMC10315140

[109] Vande Walle L Van Opdenbosch N Jacques P Fossoul A Verheugen E Vogel P . Negative regulation of the nlrp3 inflammasome by A20 protects against arthritis. Nature. (2014) 512:69–73. doi: 10.1038/nature13322, PMID: 25043000 PMC4126806

[110] Lu X Rudemiller NP Wen Y Ren J Hammer GE Griffiths R . A20 in myeloid cells protects against hypertension by inhibiting dendritic cell-mediated T-cell activation. Circ Res. (2019) 125:1055–66. doi: 10.1161/circresaha.119.315343, PMID: 31630621 PMC7006714

[111] Wenink MH Santegoets KC Butcher J van Bon L Lamers-Karnebeek FG van den Berg WB . Impaired dendritic cell proinflammatory cytokine production in psoriatic arthritis. Arthritis rheumatism. (2011) 63:3313–22. doi: 10.1002/art.30577, PMID: 21811995

[112] Moujalled DM Cook WD Okamoto T Murphy J Lawlor KE Vince JE . Tnf can activate ripk3 and cause programmed necrosis in the absence of ripk1. Cell Death Dis. (2013) 4:e465–e. doi: 10.1038/cddis.2012.201, PMID: 23328672 PMC3563989

[113] Duong BH Onizawa M Oses-Prieto JA Advincula R Burlingame A Malynn BA . A20 restricts ubiquitination of pro-interleukin-1β Protein complexes and suppresses nlrp3 inflammasome activity. Immunity. (2015) 42:55–67. doi: 10.1016/j.immuni.2014.12.031, PMID: 25607459 PMC4302274

[114] Lee MJ Lim E Mun S Bae S Murata K Ivashkiv LB . Intravenous immunoglobulin (Ivig) attenuates tnf-induced pathologic bone resorption and suppresses osteoclastogenesis by inducing A20 expression. J Cell Physiol. (2016) 231:449–58. doi: 10.1002/jcp.25091, PMID: 26189496 PMC4779648

[115] Wang X Deckert M Xuan NT Nishanth G Just S Waisman A . Astrocytic A20 ameliorates experimental autoimmune encephalomyelitis by inhibiting nf-κb- and stat1-dependent chemokine production in astrocytes. Acta Neuropathol. (2013) 126:711–24. doi: 10.1007/s00401-013-1183-9, PMID: 24077734

[116] McLornan DP Pope JE Gotlib J Harrison CN . Current and future status of jak inhibitors. Lancet. (2021) 398:803–16. doi: 10.1016/S0140-6736(21)00438-4, PMID: 34454676

[117] Kondo N Kuroda T Kobayashi D . Cytokine networks in the pathogenesis of rheumatoid arthritis. Int J Mol Sci. (2021) 22:10922. doi: 10.3390/ijms222010922, PMID: 34681582 PMC8539723

[118] Tanaka Y Luo Y O’Shea JJ Nakayamada S . Janus kinase-targeting therapies in rheumatology: A mechanisms-based approach. Nat Rev Rheumatol. (2022) 18:133–45. doi: 10.1038/s41584-021-00726-8, PMID: 34987201 PMC8730299

[119] McInnes IB Anderson JK Magrey M Merola JF Liu Y Kishimoto M . Trial of upadacitinib and adalimumab for psoriatic arthritis. New Engl J OF Med. (2021) 384:1227–39. doi: 10.1056/NEJMoa2022516, PMID: 33789011

[120] Ghoreschi K Balato A Enerback C Sabat R . Therapeutics targeting the il-23 and il-17 pathway in psoriasis. Lancet. (2021) 397:754–66. doi: 10.1016/s0140-6736(21)00184-7, PMID: 33515492

[121] Chimenti MS Latini A Conigliaro P Triggianese P Greco E De Benedittis G . Traf3ip2, hcp5 and il10 genes polymorphisms influence the response to tnf-I in patients with psoriatic arthritis. Ann Rheumatic Dis. (2022) 81:841–2. doi: 10.1136/annrheumdis-2022-eular.2446

[122] Wampler Muskardin T Vashisht P Dorschner JM Jensen MA Chrabot BS Kern M . Increased pretreatment serum ifn-β/α Ratio predicts non-response to tumor necrosis factor α Inhibition in rheumatoid arthritis. Ann rheumatic Dis. (2016) 75:1757–62. doi: 10.1136/annrheumdis-2015-208001, PMID: 26546586 PMC4860184

[123] Merola JF Landewe R McInnes IB Mease PJ Ritchlin CT Tanaka Y . Bimekizumab in patients with active psoriatic arthritis and previous inadequate response or intolerance to tumor necrosis factor-α Inhibitors: A randomized, double-blind, placebo-controlled, phase 3 trial (Be complete). Lancet. (2023) 401:38–48. doi: 10.1016/s0140-6736(22)02303-0, PMID: 36495881

[124] Urbano PCM Aguirre-Gamboa R Ashikov A van Heeswijk B Krippner-Heidenreich A Tijssen H . Tnf-α-induced protein 3 (Tnfaip3)/A20 acts as a master switch in tnf-α Blockade-driven il-17a expression. J Allergy Clin Immunol. (2018) 142:517–29. doi: 10.1016/j.jaci.2017.11.024, PMID: 29248493

[125] Urbano PCM He X van Heeswijk B Filho OPS Tijssen H Smeets RL . Tnfα-signaling modulates the kinase activity of human effector treg and regulates il-17a expression. Front Immunol. (2019) 10:3047. doi: 10.3389/fimmu.2019.03047, PMID: 32038615 PMC6986271

